# A recovery principle provides insight into auxin pattern control in the Arabidopsis root

**DOI:** 10.1038/srep43004

**Published:** 2017-02-21

**Authors:** Simon Moore, Junli Liu, Xiaoxian Zhang, Keith Lindsey

**Affiliations:** 1 Department of Biosciences, Durham University, South Road, Durham DH1 3LE, UK; 2 Department of Sustainable Soil and Grassland System, Rothamsted Research, Harpenden, Hertfordshire AL5 2GQ, UK

## Abstract

Regulated auxin patterning provides a key mechanism for controlling root growth and development. We have developed a data-driven mechanistic model using realistic root geometry and formulated a principle to theoretically investigate quantitative auxin pattern recovery following auxin transport perturbation. This principle reveals that auxin patterning is potentially controlled by multiple combinations of interlinked levels and localisation of influx and efflux carriers. We demonstrate that (1) when efflux carriers maintain polarity but change levels, maintaining the same auxin pattern requires non-uniform and polar distribution of influx carriers; (2) the emergence of the same auxin pattern, from different levels of influx carriers with the same nonpolar localisation, requires simultaneous modulation of efflux carrier level and polarity; and (3) multiple patterns of influx and efflux carriers for maintaining an auxin pattern do not have spatially proportional correlation. This reveals that auxin pattern formation requires coordination between influx and efflux carriers. We further show that the model makes various predictions that can be experimentally validated.

A major challenge in plant developmental biology is understanding how development is coordinated by interacting hormones and genes. The regulated formation of auxin gradients provides a key mechanism controlling plant growth, tropisms and development through the provision of positional and vectorial information^1^. Arabidopsis root development is coordinated via an auxin concentration maximum in the root tip^2^. The auxin maximum specifies the hypophysis and quiescent centre (QC), regulates root meristem formation, and positions the stem cell niche (SCN)^2^. An auxin minimum also defines a developmental window for lateral root initiation^3^, while transport of auxin produced in new lateral root primordia regulates lateral root emergence^4^. Low rates of polar auxin transport balances cell differentiation and division and prevents meristem growth, while high polar auxin transport promotes cell division over differentiation^5^. These and many other studies show that understanding the quantitative properties of auxin patterning is essential for understanding the regulation of root development.

Auxin gradient formation in the Arabidopsis root is predominantly regulated by auxin transport proteins^6^, including PIN-FORMED (PIN) proteins^7^, the AUX1/LIKE-AUX1 (AUX1/ LAX) family of influx carriers/channels^8^, and ABCB transporters^9^,^10^. Auxin gradients are hypothesized to be sink-driven^11^, while modelling has suggested that PIN efflux carriers can generate the gradient^12^. Other studies indicate that AUX1/LAX influx carriers are also required for creating auxin distribution patterning at the root tip^13^,^14^. Importantly, it has been suggested that nonpolar AUX1/LAX influx carriers create tissues containing high auxin concentrations, while polar PIN carriers control directional auxin transport within these tissues^13^. Therefore both auxin efflux^12^,^15^,^16^ and influx carriers^13^,^16^ are considered important for generating auxin patterning, although their relative contributions are not clear.

While it is known that polar PIN carriers direct auxin movement differentially and nonpolar AUX1/LAX carriers act to retain cellular auxin, one key question is how the combined transport activity of the polar PIN and nonpolar AUX1/LAX carriers can potentially control auxin pattern formation. Important auxin carrier properties include their concentration and localisation. Concentration is controlled by gene expression and protein turnover, and localisation by polar or nonpolar recycling and distribution at plasma membranes^7^,^8^. One critical component of understanding auxin pattern formation requires investigation of how concentration and localisation of influx and efflux carriers potentially work together to generate pattern. The following questions need to be addressed. First, while maintaining constant nonpolar AUX1/LAX localisation but at different levels, can auxin patterning be maintained without changing PIN polarity? Similarly, while maintaining polar PIN localisation but at different levels, does auxin pattern maintenance require changes in AUX1/LAX localisation? Answers will determine how influx and efflux carrier levels and localisation combine to control auxin pattern formation and clarify the individual roles of polar PIN and nonpolar AUX1/LAX carriers in maintaining auxin patterning. Second, can auxin pattern recovery lead to auxin carrier pattern recovery? Answers will address the role of auxin in regulating the patterning of its own transporters. Third, for the same auxin pattern generated by different influx and efflux carrier combinations, are the influx and efflux carrier levels spatially correlated? Answers will reveal how auxin patterning is controlled by combined influx and efflux carrier patterning.

This study seeks answers through data-driven mechanistic modelling analysis, in which we explicitly include the polar PIN and nonpolar AUX1/LAX carriers. Since the ABCB family can reversibly redirect auxin flux^9^,^10^, the role of these transporters has been incorporated into PIN and AUX1/LAX activity to simplify modelling analysis. Our model integrates the following experimental data: (1) a root structure with cell geometries derived from confocal microscopy imaging^13^, where each cell has a cytosolic space, plasma membrane and cell wall; (2) PIN and AUX1/LAX carrier localisation based on experimental images^11^,^13^,^17^,^18^,^19^,^20^; (3) PIN polarity; and (4) experimental data describing hormonal crosstalk between efflux carriers (PIN1 and PIN2) and hormones (auxin, ethylene, cytokinin)^21^. The hormonal crosstalk network is a mixed-type network that integrates gene expression, signal transduction and metabolic conversions. In particular, the important processes related to auxin patterning incorporated in the network include (a) auxin biosynthesis and degradation; (b) auxin transport facilitated by both influx and efflux carriers; and (c) regulatory relationships^21^. Therefore, the network integrates auxin metabolism and transport into an integrative system.

We show that a model integrating the above data reproduces auxin patterning similar to experimental observations. We also formulate a general principle for quantitative auxin pattern recovery which demonstrates how relationships between influx and efflux carrier level and localisation can possibly combine to quantitatively control auxin patterning and the emergence of specific auxin patterns, which demonstrates that the relationship between influx and efflux carriers, not their individual activity, regulates auxin patterning. This principle allows relationships between level and localisation of influx and efflux carriers for a target auxin pattern to become searchable, and it enables auxin concentration and its influx and efflux carriers to be studied as an integrated system. In addition, we show that our model makes various predictions that can be validated experimentally.

## Results

### Data-driven mechanistic modelling reproduces the key features of auxin response patterning in root development

**Figure 1:**
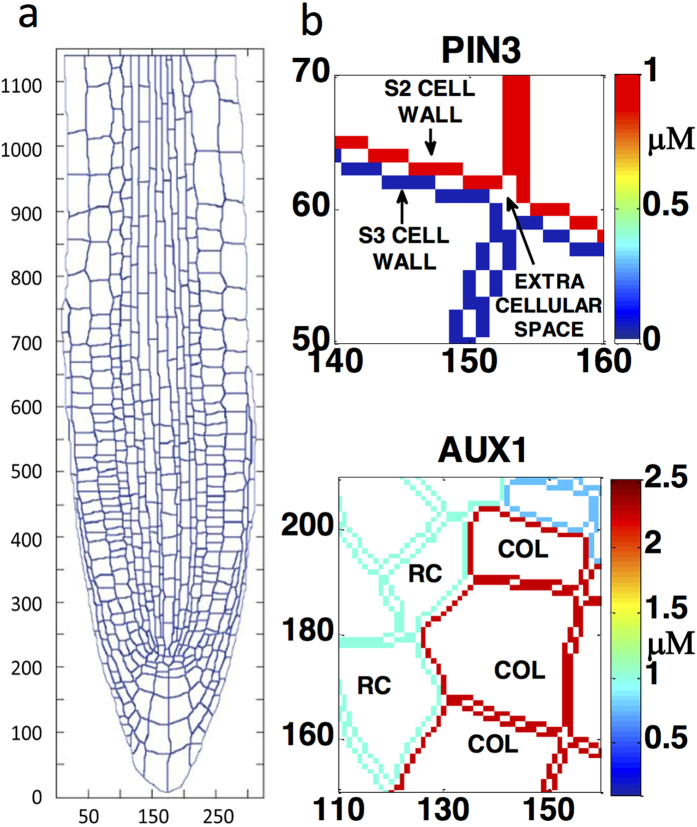
The model root with realistic cell geometry, a cytosolic space for each cell, a unique combined plasma membrane and cell wall entity for each cell, extracellular space, and auxin influx and efflux carrier localisation. (**a**) A realistic root map showing the individual cells, based on confocal imaging^13^. (**b**) Localisation of efflux (PIN3) and influx (AUX1) carriers at the combined plasma membrane and cell wall entity of selected cells, with extra-cellular space between the cell walls of adjacent cells. (S2 and S3: columella tier 2 and 3 cells. COL: columella, RC: root cap). The details of the data-driven mechanistic model are included in the Supplementary Methods.

Here we integrate experimental data into a systematic modelling analysis of auxin patterning. Figure 1 and Supplementary Methods describe a multicellular hormonal crosstalk model with realistic cell geometries, localisation of auxin influx and efflux carriers, and PIN carrier polarity. Our model (Fig. 1) integrates four categories of experimental data, as follows and as described in Supplementary Methods.

First, actual cell geometry and multicellular root organisation allows the study of cell-cell communication. Each cell contains two spatial identities, the cytosolic space, and a space including the plasma membrane and cell wall. For simplicity, adjacent plasma membrane and cell wall entities are represented by a single model identity containing both cell wall and plasma membrane properties. We refer to this single identity as either a cell wall or plasma membrane depending on the context and properties under discussion. We also consider extracellular space but do not include any subcellular structures. Auxin is transported between the two spatial identities by influx and efflux carriers, while within the cytosol or within the combined cell wall and extracellular space, auxin is considered to be transported by diffusion. At the shoot-root boundary, conditions for auxin concentration are fixed and implemented in the same way as described in the literature^21^.

Second, localisation of PIN efflux (PIN1,2,3,4,7) and AUX1/LAX influx carriers (AUX1, LAX2,3) at the plasma membrane is derived from experimental imaging and implicitly includes ABCB transporter activity (Supplementary Methods). Since the ABCB family can reversibly redirect auxin flux, we consider that ABCB activity is incorporated into the basal activity of all PIN proteins and AUX1/LAX influx carriers. These basal activities for both efflux and influx carriers are nonpolar. Since PIN proteins can have polarised distribution, we consider that the lowest levels of PIN proteins have included the activities of PIN and ABCB proteins. For example, the lowest concentration of PIN3 protein is prescribed to be 0.06 μΜ (Supplementary Methods). We consider this concentration has implicitly included the activities ABCB proteins (say 0.02 μΜ is attributed to the ABCB protein activity). Since PIN3 distribution is polarised, we consider that the areas with higher PIN3 concentrations have also included the same ABCB protein activity.

Third, PIN (PIN1,2,3,4,7) carrier polarity is defined based on experimental data (Supplementary Methods); and fourth, experimental data on the hormonal crosstalk network are as constructed previously^21^,^22^,^23^; and as summarised in Supplementary Methods.

In the cytosolic spaces, the crosstalk network controls metabolism of two efflux carriers (PIN1 and PIN2) and the hormones auxin, ethylene and cytokinin (Supplementary Methods). The network allows quantitative description of PIN1 and PIN2 regulation by the three hormones and enables study of the relationship between auxin, PIN1 and PIN2 patterning. The crosstalk networks for other auxin carriers (PIN3,4,7, AUX1, LAX2,3) cannot be constructed due to insufficient experimental data, as analysed in Supplementary Methods, and therefore these carriers are prescribed based on experimental imaging.

Kinetic equations and parameters for crosstalk between PIN1, PIN2, auxin, ethylene and cytokinin are as previously described^21^. Concentration levels for PIN3,4,7 and AUX1, LAX2,3 are adjusted so that the model reproduces key features of wildtype auxin patterning (Supplementary methods).

**Figure 2:**
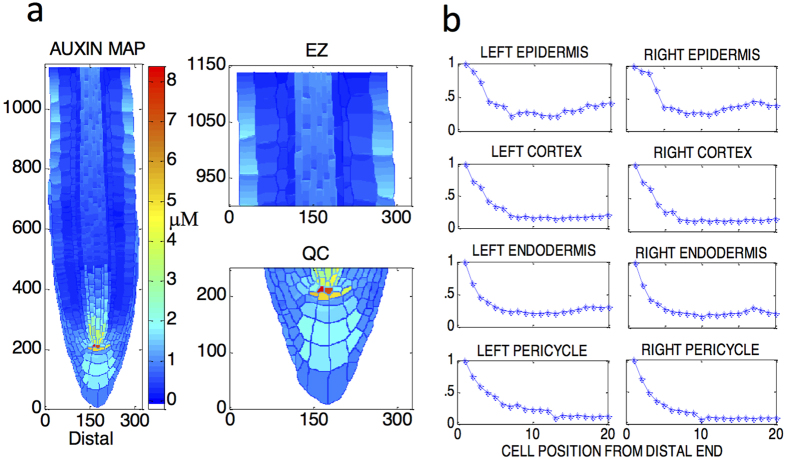
Modelled auxin concentration patterning and relative auxin concentration trends above the initials for selected cell files. (**a**) Auxin concentration colour map for the root and selected regions. (**b**) Relative auxin concentration trends in the first 20 cells above the initials in the epidermis, cortex, endodermis and pericycle cell files. (EZ: Elongation zone. QC: Quiescent Centre).

The model reproduces key features of experimental auxin response patterning (Fig. 2). Specifically, an auxin maximum emerges at the quiescent centre (QC); auxin concentration in epidermal cells in the elongation zone is higher than that in the pericycle and vascular cells as observed experimentally^13^; a steep auxin gradient is observed in cells closest to the QC starting at the initials, similar to experimental R2D2 reporter gradients (Fig. 2 and Figure S6 in Liao *et al*.^24^). Auxin distribution in different cell types is also comparable with experimental DII-VENUS reporter data (Fig. 1K in Band *et al*.^13^; Figure S1). The trend of the modelled auxin concentrations for five cell types (i.e. QC, stele, endodermis, epidermis meristem and cortex meristem) is similar to the trend observed experimentally (Fig. 1K in Band *et al*.^13^). The discrepancies between our modelling results (Figure S1) and the experimental observations of DII-VENUS response (Fig. 1K in Band *et al*.^13^) were similarly observed in the previous modelling results where a rectangular root structure was used (Notes S1 in Moore *et al*.^21^). The possible reasons for these discrepancies were discussed comprehensively in our previous research (Notes S1 in Moore *et al*.^21^).

The model also predicts that PIN1 and 2 levels generally decrease from the proximal to distal region of the root (Figure S1), similar to experimental observations (Fig. 1 in Liu *et al*.^23^; Fig. 3B in Bishopp *et al*.^25^; Fig. 5 in Rowe *et al*.^26^). Therefore experimental data can be integrated into a systems model which is able to reproduce key features of auxin patterning and predict PIN1 and 2 patterning in the root (Figs 1, 2 and S1).

### The interlinked relationship between auxin concentration and influx and efflux carrier patterning

Since the model integrates a wide range of experimental data and reproduces key features of auxin response patterning, we can investigate how the combined activities of PIN and AUX1/LAX carriers can potentially control auxin pattern formation, provided we can develop a general principle for quantitative auxin pattern recovery following perturbation of transporter activity. Such a principle will reveal how specific auxin patterning can be generated by the relationship between influx and efflux carriers, rather than the activities of individual carrier types.

#### A principle for the quantitative recovery of an auxin pattern: ‘Recovery Principle’

In a multicellular root with realistic cell geometry, cells may exchange auxin with different numbers of neighbouring cells (Fig. 1). Although polar PIN carriers direct auxin in specific directions and nonpolar AUX1/LAX carriers act to retain cellular auxin, an important question is whether PIN and AUX1/LAX carriers can work together to maintain a specific auxin pattern. To address this, we derive a mechanism for quantitative auxin pattern recovery, termed the ‘recovery principle’.

The principle is an iterative process designed to study steady-state patterning. Therefore, each iteration must be computed for a sufficiently long simulation time for all components to reach steady state values.

Since AUX1/LAX influx carriers direct auxin from the cell wall to the cytosol, a recovery principle after PIN carrier perturbation can be formulated as follows.


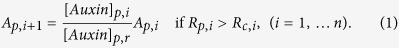



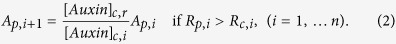


With


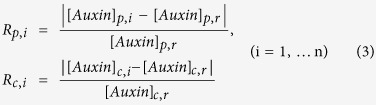


*A*_*p*,i+1_ is the searched ‘new’ AUX1/LAX concentration, calculated by adjusting the ‘old’ AUX1/LAX concentration (*A*_*p*,i_) using the above equations. At the first iteration (i = 1), *A*_*p*,*i*_ is equal to the AUX1/LAX concentration in the reference system (e.g. wildtype), (*A*_*p*,*r*_). [*Auxin*]_*p*,*i*_ is the auxin concentration at the cell wall gridpoint (adjacent to one or more cytosolic gridpoints). [*Auxin*]_*c*,*i*_ is the auxin concentration at a cytosolic gridpoint connecting to one or more adjacent cell wall gridpoints. At the first iteration (i = 1), [*Auxin*]_*c*,*i*_ is the auxin concentration when *A*_*p*,*i*_ equals *A*_*p*,*r*_ and PIN carrier concentrations equal their values after initial perturbation from their reference state. [*Auxin*]_*c*,*r*_ and [*Auxin*]_*p*,*r*_ are the respective auxin concentrations of a reference system at a cytosolic and cell wall gridpoint, respectively. *R*_*p*,*i*_ and *R*_*c*,*I*_ are the absolute values of the relative differences in auxin concentration between the current and reference systems at the cell wall and cytosolic gridpoints for each iteration, respectively. *R*_*p*,*i*_ and *R*_*c*,*I*_ quantify the degree to which the auxin concentration in the current system differs from the reference system. The recovery principle, eqs 1,2,3, has the following biological significances. PIN level perturbations change auxin patterning. To recover the original auxin pattern, we compare the changed auxin pattern (in the current system) with the reference pattern and then reset AUX1/LAX patterning using eqs 1,2,3. Since changing the auxin concentration at any gridpoint results in changes at all neighbouring gridpoints, a change at any location will change concentrations across the whole root. Therefore, to recover the auxin pattern, AUX1/LAX must be reset simultaneously at all root locations using eqs 1,2,3. The AUX1/LAX adjustments at each cell wall location are calculated using the auxin concentration at the cell wall or neighbouring cytosolic gridpoint which differs most from the reference value.

Specifically, when two auxin patterns are compared and if *R*_*p*,*i*_ > *R*_*c*,*i*_ for i = 1…n, the AUX1/LAX activity should be changed so that cell wall auxin concentration is adjusted towards its reference value. To do this, AUX1/LAX concentration should change by a factor of 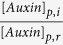 for i = 1…n, following equation 1. For example if 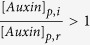 for i = 1…n, total AUX1/LAX concentration increases, resulting in a decrease in auxin concentration at the cell wall gridpoint which in turn results in increased auxin concentration at the neighbouring cytosolic gridpoint. Similarly, if *R*_*p*,*i*_ ≤ *R*_*c*,*i*_ for i = 1…n, AUX1/LAX activity should be changed so that auxin concentration at the cytosolic gridpoint changes towards its reference value. To do this, total AUX1/LAX concentration should change by a factor of 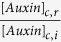 for i = 1…n, following equation 2.

Thus, after all gridpoints in the whole root are compared simultaneously to the reference system, a new AUX1/LAX concentration pattern is created for the whole root. The new AUX1/LAX concentration pattern generates a new auxin pattern which is again compared with the reference pattern, following the recovery principle. As a result, the auxin pattern gradually approaches the reference pattern. After multiple iterations, the original reference auxin pattern is recovered.

Therefore, the recovery principle is a generic, biologically based, iterative relationship that establishes how auxin efflux and influx carriers can coordinate their activities to control the emergence of a specific auxin pattern.

**Figure 3:**
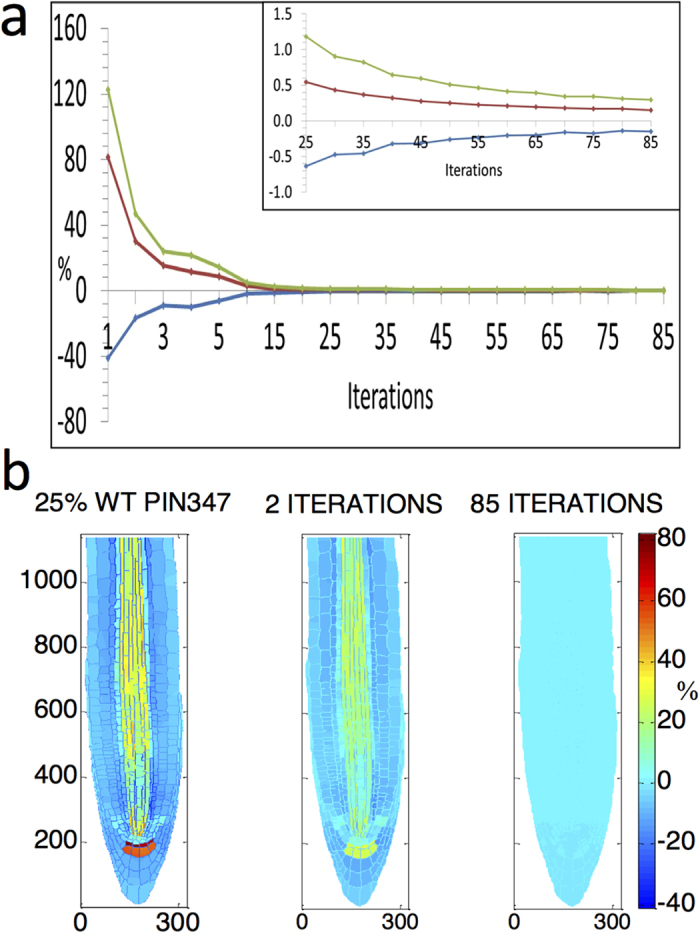
Auxin pattern recovery after 75% decrease in total wildtype PIN3,4,7 concentrations, using the recovery principle over 85 iterations. (**a**) Progressive auxin percentage difference from wildtype in the whole root. Blue curve shows the maximum auxin percentage difference below wildtype in the root, at each iteration. Red curve shows the maximum auxin percentage difference above wildtype in the root, at each iteration. Green curve shows the maximum range of the percentage difference from wildtype within the root, calculated by adding the absolute values of the blue and red curves at each iteration. (**b**) Colour map images of the percentage difference from wildtype auxin concentrations in the root after the initial perturbation of 75% loss of PIN3,4,7, then after 2 recovery iterations, and at full recovery after 85 iterations. Symbol % is the percentage difference relative to corresponding wildtype value.

Figure 3 presents an example of auxin pattern recovery after total PIN3, 4 and 7 concentration is decreased by 75% of its wildtype reference value.

After reducing total PIN3, 4 and 7 by 75%, the maximum deviation in auxin concentration from reference is ca. 80% across the whole root (Fig. 3). Using the recovery principle to reset AUX1/LAX, this difference reduces to ca. 10% after 5 iterations (Fig. 3). After 85 iterations it reduces to ca. 0.3%, indicating that wildtype root auxin patterning is fully recovered (Fig. 3). Therefore after a 75% PIN3, 4, 7 reduction, wildtype auxin patterning can be maintained by resetting AUX1/LAX following the recovery principle. This indicates that the relationship between influx and efflux carriers, rather than their individual activities, controls auxin pattern formation.

We have examined additional five different total PIN3, 4 and 7 concentrations set at 0%, 50%, 150%, 175% and 200% of wildtype, finding that the recovery principle can always recover a reference wildtype auxin pattern (Figures S2–S6). Therefore, it is clear that wildtype auxin patterning can emerge from multiple combinations of interlinked levels and localisation of influx and efflux carriers. Thus, PIN and AUX1/LAX influx carriers in combination control auxin pattern formation.

Auxin pattern recovery also leads to quantitative recovery of PIN1 and 2 patterning (Figure S7). After reducing total PIN3, 4 and 7 concentration, modelling results predict that PIN1 and 2 concentration increases in the plasma membrane of vascular cells (Figure S7). This is similar to experimental observations for the *pin3pin4pin7* triple mutant^18^. Although the level and patterning of both total PIN3,4,7 and AUX1/LAX differ from their original patterning after auxin recovery, PIN1 and 2 levels and patterning return to their original values. This reflects the regulation of PIN1 and 2 levels and patterning by hormonal crosstalk and demonstrates the mutual regulation of auxin and its transporters via hormonal crosstalk. Therefore, the recovery principle reveals that the patterning of auxin concentration and the influx and efflux carriers is interlinked.

The recovery principle can also be used to search for the level and localisation of PIN efflux carriers necessary to recover AUX1/LAX perturbations, as summarised in section “Quantitative recovery of an auxin pattern requires changes in PIN carrier polarity when AUX1/LAX influx carriers maintain uniform distribution but change levels”, below.

#### When PIN efflux carriers maintain polarity but change levels, quantitative recovery of an auxin pattern requires non-uniform and polar distribution of AUX1/LAX influx carriers

**Figure 4:**
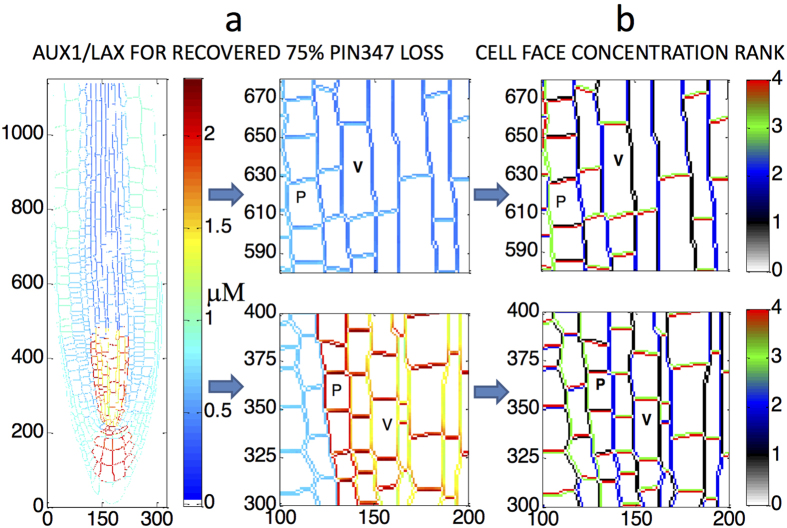
AUX1/LAX concentration patterning required for auxin pattern recovery after reducing total PIN3, 4 and 7 by 75%. (**a**) AUX1/LAX colour map for recovery requires non-uniform and polar distribution of AUX1/LAX. (**b**) AUX1/LAX average cell face concentrations are ranked 1 to 4 for each cell, showing polar distribution of AUX1/LAX influx carriers. This is calculated by averaging the data in each cell face in (**a**) and by ranking them in terms of the average values. P: pericycle cell, V: vascular cell.

The computed AUX1/LAX carrier patterns for wildtype auxin pattern recovery reveal that, when PIN carriers maintain their polarity but change levels, auxin pattern recovery requires non-uniform and polar distribution of AUX1/LAX (Fig. 4). AUX1/LAX concentrations can vary at the same cell face and AUX1/LAX influx carrier polarity can also be identified. In recovery from 75% loss of PIN3, 4 and 7, regions of vascular and pericycle cells exhibit higher AUX1/LAX levels at apical cell faces (Fig. 4). This polarity can be similar or opposite to PIN polarity depending on root location and the original perturbation (Figs 4 and S8).

Experimentally, AUX1/LAX appears to have nonpolar distribution^13^,^27^. In the wildtype, polar PIN and nonpolar AUX1/LAX carriers combine to generate wildtype auxin patterning. When total wildtype PIN3, 4 and 7 is perturbed, wildtype auxin patterning requires non-uniform and polar distribution of AUX1/LAX influx carriers, which highlights the importance of combined polar PIN and nonpolar AUX1/LAX carrier activity in generating a specific quantitative auxin pattern.

#### A uniform distribution of AUX1/LAX influx carriers leads to changes in the recovered auxin pattern

**Figure 5:**
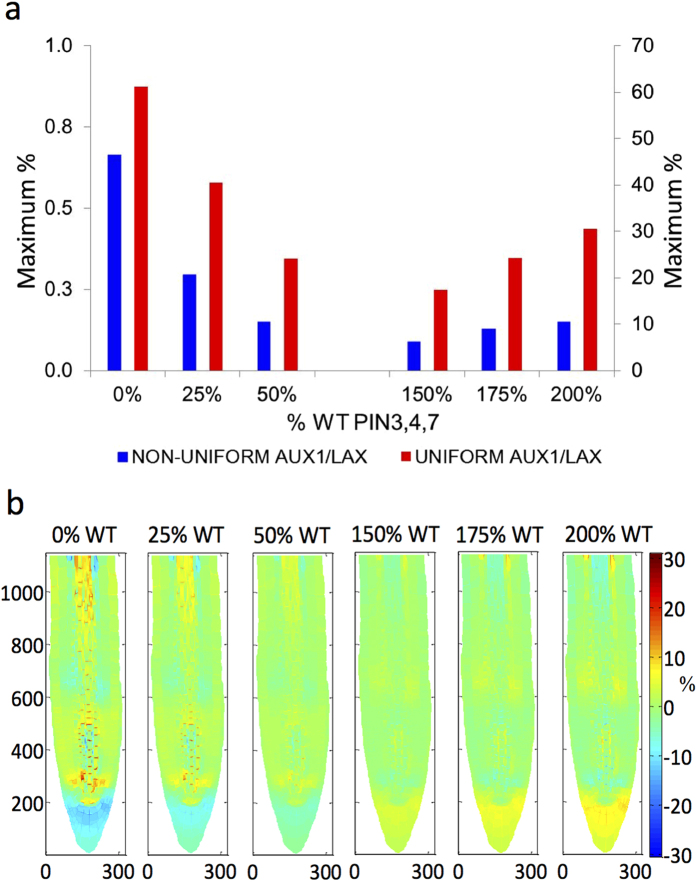
Comparison of auxin pattern recovery by uniform and non-uniform distribution of AUX1/LAX, for 6 different total PIN3, 4 and 7 concentrations set at 0%, 25%, 50%, 150%, 175% and 200% of wildtype. (**a**) Maximum percentage difference of auxin concentration from WT after recovery by uniform (red bar, right y-axis) and non-uniform (blue bar, left y-axis) AUX1/LAX distribution, for each case. (**b**) Colour maps of percentage difference of auxin concentration from WT after recovery by uniform AUX1/LAX distribution, for each case. Symbol % is the percentage difference relative to corresponding wildtype value.

To further explore the implications of PIN and AUX1/LAX carriers in generating an auxin pattern, we averaged AUX1/LAX concentrations over the plasma membrane of each cell in the root for the six recovery cases investigated. Although averaging does not change the total AUX1/LAX level in a cell, it guarantees a uniform AUX1/LAX distribution at the plasma membrane of each cell. After averaging, AUX1/LAX carriers have uniform cellular distribution, but the average varies between cells. Figure 5 shows that uniform AUX1/LAX distribution leads to changes in auxin patterning compared to the equivalent non-uniform AUX1/LAX distribution. This demonstrates that, while non-uniform polar AUX1/LAX distribution can recover wildtype auxin patterning, the corresponding uniform distribution cannot. Therefore, when the same level of AUX1/LAX influx carriers is maintained, uniform distribution generates a specific auxin pattern that is different from the auxin patterns generated by non-uniform distribution, indicating the importance of uniform distribution of AUX1/LAX influx carriers in the control of auxin patterning.

#### Patterns of PIN and AUX1/LAX carriers for maintaining an auxin pattern do not have spatially proportional correlation

We investigated six cases for recovering wildtype auxin patterning and found that multiple combinations of interlinked PIN and AUX1/LAX patterns can lead to the same target auxin pattern. Biologically, polar PIN carriers differentially direct auxin, while AUX1/LAX carriers act to retain cellular auxin. For further insights into how PIN and AUX1/LAX carriers can combine to generate specific auxin patterning, we explored whether multiple combinations of interlinked PIN and AUX1/LAX patterns for generating the same auxin pattern exhibit spatially proportional correlation. Any correlation would imply that the effect on auxin patterning of changing polar PIN activity can be compensated by proportionately changing AUX1/LAX activity.

Figure S9 reveals the spatial complexity of relationships between influx and efflux carriers. Different PIN and AUX1/LAX pattern combinations, that maintain the same auxin pattern, do not exhibit spatially proportional correlation. Although auxin patterning is recovered, the ratio of PIN between 75% PIN3,4,7 reduction and wildtype is generally not equal to the corresponding ratio for AUX1/LAX. This reveals that the effects on auxin patterning of changing polar PIN activity are not compensated by a proportional change in AUX1/LAX activity. Another level of complexity is that relationships between PIN and AUX1/LAX for maintaining an auxin pattern depend on the absolute levels of these carriers. Comparing Figure S9a and b shows that 75% reduction of PIN3,4,7 and 75% increase of PIN3,4,7 require different relationships between PIN and AUX1/LAX to maintain the same auxin patterning. This complexity exists for all six cases investigated (total PIN3, 4 and 7 concentrations are set at 0%, 25%, 50%, 150%, 175% and 200% of wildtype) (Figures S9, S10 and S11). Therefore, PIN and AUX1/LAX patterning for maintaining an auxin pattern do not have a spatially proportional correlation.

#### Quantitative recovery of an auxin pattern requires changes in PIN carrier polarity when AUX1/LAX influx carriers maintain uniform distribution but change levels

At different PIN levels, maintaining an auxin pattern can require non-uniform and polar AUX1/LAX distribution (Fig. 4). A uniform AUX1/LAX distribution leads to deviations from target auxin patterning (Fig. 5). This highlights the importance of polar PIN and nonpolar AUX1/LAX carrier combinations in generating a specific auxin pattern.

The recovery principle can also be used to search for the level and localisation of PIN when AUX1/LAX carriers are perturbed, by considering the role of PIN carriers in auxin transport from the cytosolic to cell wall. As such, the recovery principle requires that equations 1 and 2 be replaced by equations 4 and 5.


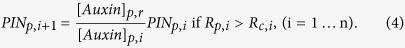



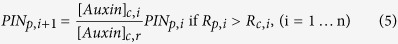


*PIN*_*p*,*i*+1_ is the searched ‘new’ PIN concentration, calculated by adjusting the ‘old’ PIN concentration, *PIN*_*p*,*i*_, using the above equations. At the first iteration (i = 1), *PIN*_*p*,*i*_ is the PIN concentration in the reference system.

**Figure 6:**
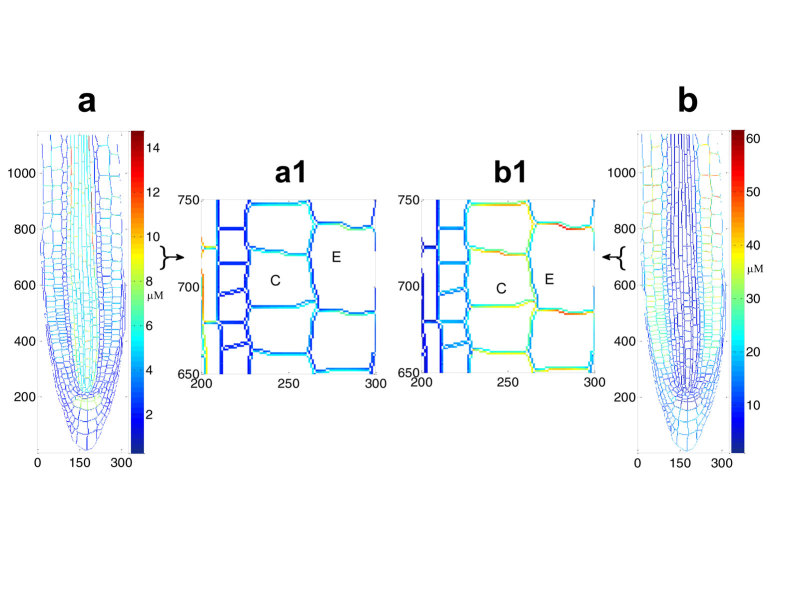
The polarity of PIN3,4,7, after wildtype auxin pattern recovery from a 50% gain in AUX1/LAX level, is different from PIN3,4,7 polarity in wildtype. (**a**) Colour map of total PIN3,4,7 after recovery to wildtype auxin patterning. a1 shows an enlarged view of this map in selected epidermal and cortical cells. (**b**) Colour map of the ratio of PIN3,4,7 after auxin pattern recovery to wildtype PIN3,4,7. b1 shows enlarged views of this map in the epidermal and cortical cells, corresponding to the regions shown in a1. PIN3,4,7 have no polarity in the wildtype epidermal and cortical cell files (see Fig. 3 in the Supplementary Methods), however PIN3,4,7 polarity can be observed in these cells after recovery, in a1, where there is greater PIN3,4,7 concentrations at the apical cell faces. This is confirmed in b1 where the red/brown/yellow colours indicate a consistently larger percentage increase in PIN3,4,7 after recovery compared to wildtype at the apical faces of these cells. This result shows that, in these epidermal and cortical cells, the polarity of PIN3,4,7, after wildtype auxin pattern recovery from a 50% gain in AUX1/LAX level, is different from PIN3,4,7 polarity in wildtype. C: cortical cells. E: epidermal cells.

The recovery principle shows that, at different levels of uniformly localised AUX1/LAX, maintenance of auxin pattern requires changes in PIN polarity (Fig. 6).

When AUX1/LAX levels are increased to 50% above wildtype, wildtype auxin patterning can be recovered, but the searched PIN3,4,7 polarity differs from wildtype. The searched total PIN3,4,7 localisation in epidermal cells displays polarity that does not exist in wildtype (Fig. 6), indicating that the corresponding relationship between influx level and efflux polarity, rather than the individual activity of either the influx or efflux carriers, controls auxin pattern formation.

The recovery principle reveals that the same auxin pattern can emerge from multiple combinations of interlinked, but not spatially proportionally correlated, levels and localisation of both influx and efflux carriers. Specifically, at different levels of efflux carriers with the same polarity, both the level and polarity of influx carriers must be simultaneously changed to maintain auxin patterning. At different levels of influx carriers with the same nonpolar localisation, both the level and polarity of efflux carriers require simultaneous adjustment for auxin pattern maintenance. In addition, a recovered auxin pattern leads to quantitative PIN1 and 2 pattern recovery. Therefore, auxin concentration, and influx and efflux carrier patterning are interlinked into an integrated system.

### The recovery principle enables searchable relationships between level and localisation of both influx and efflux carriers for a known target auxin pattern

**Figure 7:**
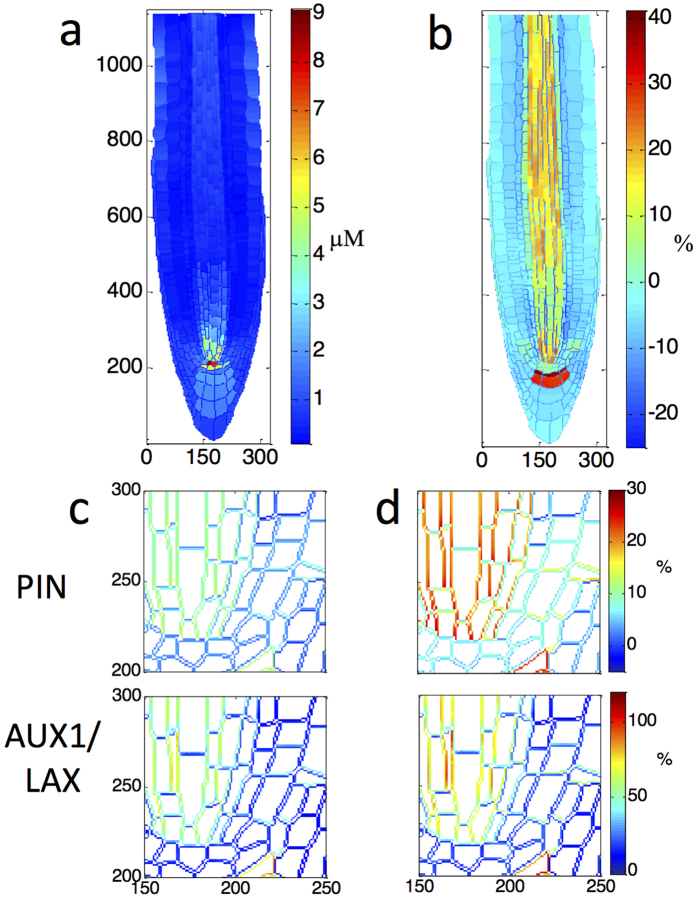
Recovery to the same target auxin pattern from a 15% gain in PIN3,4,7 and from a 30% gain in PIN3,4,7, shows that two very different combinations of interlinked PIN and AUX1/LAX can lead to the same auxin pattern. (**a**) Auxin concentration colour map for the target auxin pattern created by a 50% loss in PIN3,4,7. (**b**) The percentage difference of target auxin pattern from the WT. (**c**) Total PIN and AUX1/LAX percentage comparison to WT for recovery from a 15% gain in PIN3,4,7, for a selected area of the root. (**d**) Total PIN and AUX1/LAX percentage comparison to WT for recovery from a 30% gain in PIN3,4,7, for the same selected area of the root. In (**c**,**d**), symbol % is the percentage difference relative to corresponding wildtype value.

The recovery principle reveals that the same wildtype reference auxin pattern can emerge from multiple combinations of interlinked, but not spatially proportionally correlated, levels and localisation of influx and efflux carriers. This principle unravels novel aspects of auxin patterning control and enables the search for unknown PIN and AUX1/LAX carrier combinations, that generate a known target auxin pattern (Fig. 7).

The recovery principle identifies two interlinked PIN and AUX1/LAX patterns (Fig. 7c,d) that both recover a known target auxin pattern (Fig. 7a,b). Although PIN patterning in Fig. 7c,d differ significantly, when combined with their corresponding AUX1/LAX patterns they generate the same target auxin pattern. Thus, for a known target auxin pattern, the recovery principle can search for multiple PIN and AUX1/LAX pattern combinations that achieve the target patterning. Therefore relationships between level and localisation of influx and efflux carriers become searchable.

### A systems view of the control of auxin patterning by both influx and efflux carriers

**Figure 8:**
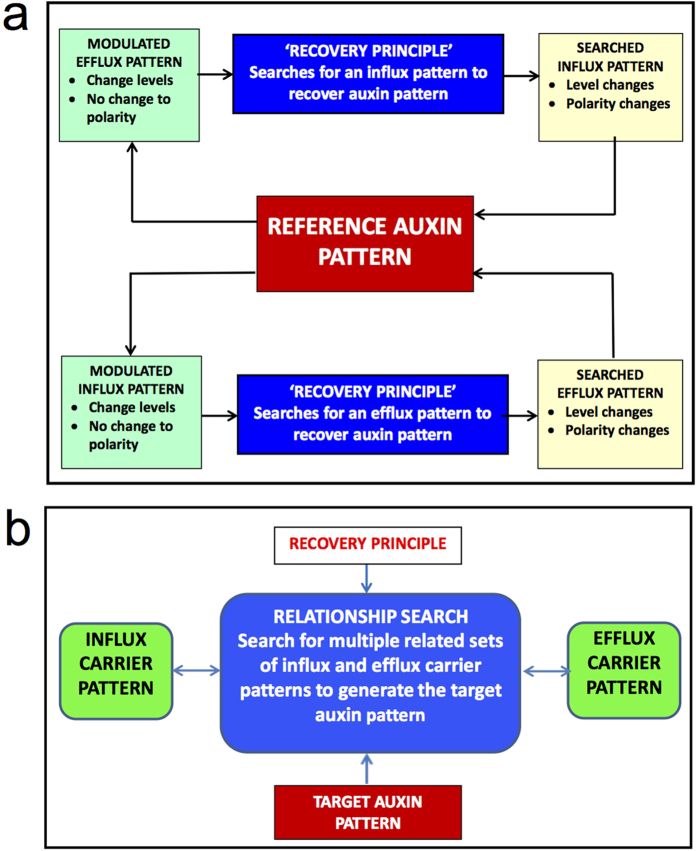
The recovery principle reveals relationships between the influx and efflux carriers in establishing auxin patterning. (**a**) Changes in level of the influx carriers require changes in both polarity and level of the efflux carriers and vice versa, for recovery. (**b**) The recovery principle makes it possible to search for influx and efflux carrier combinations that achieve a target auxin pattern.

While many factors likely control auxin patterning, the auxin influx and efflux carriers have been shown to be key components of pattern formation. The recovery principle reveals how the two important influx and efflux carrier properties, level and localisation, are potentially linked to control auxin pattern formation. Figure 8 summarises the relationships between influx and efflux carrier levels and localisation.

For maintaining a target auxin pattern, the level and localisation of influx and efflux carriers are interlinked as follows: (1) changing PIN levels and maintaining PIN polarity require changes to both AUX1/LAX levels and polarity; (2) changing AUX1/LAX levels and maintaining the nonpolar localisation requires changes to both PIN levels and polarity. Experimentally, AUX1/LAX appears to have nonpolar distribution in at least some cell types^13^,^27^. Therefore, for maintaining an auxin pattern, PIN polarity is essential, but it must change at different AUX1/LAX levels. Additionally, PIN and AUX1/LAX pattern combinations that maintain the same auxin pattern do not have a spatially proportional correlation, indicating a complex nonlinear relationship between PIN and AUX1/LAX in controlling auxin pattern formation. Therefore quantitative effects of PIN on auxin patterning must be examined concomitantly with the effects of AUX1/LAX, and vice versa.

### Model predictions and experimental validation

As described above, the model developed in this work has integrated a variety of experimental data. By formulating a recovery principle, we have shown that the model can be used to provide insights into the control of auxin patterning in the Arabidopsis root. Since the model integrates the properties of auxin influx and efflux transporters with those of auxin biosynthesis and degradation, it is also able to make various predictions that can be experimentally validated.

Figure S1 shows that the model predicts that PIN1 and 2 levels generally decrease from the proximal to distal region of the root. These predictions are similar to experimental observations (Fig. 1 in Liu *et al*.^24^; Fig. 3B in Bishopp *et al*.^25^; Fig. 5 in Rowe *et al*.^26^). This reveals that PIN1 and 2 patterning is the result of the integrative actions of a hormonal crosstalk network in the Arabidopsis root (Supplementary Methods).

Figure S12 shows modelling predictions of the combined PIN1 and PIN2 concentration patterns for 100% loss of PIN3 or PIN4 or PIN7 and for the combined 100% loss of PIN3, PIN4 and PIN7. When PIN3, PIN4 or PIN7 levels are reduced to zero, modelling results predict that the PIN1 expression domain extends further to the elongation zone. These predictions are similar to experimental observations (Fig. 6 in Omelyanchuk *et al*.^28^). In addition, after reducing total PIN3, 4 and 7 concentrations to zero, modelling results predict that PIN1 and 2 concentrations increase in the plasma membrane of vascular cells. This is similar to experimental observations for the *pin3pin4pin7* triple mutant^18^. This modelling analysis reveals that the model is able to predict changes in PIN1 patterning for other *pin* mutants. We note that these model predictions have assumed that, for a *pin* mutant, the dynamics of PIN1 and 2 is regulated by a hormonal crosstalk (Supplementary Methods) while all other auxin influx and efflux transporters remain unchanged. In this work, since both PIN1 and PIN2 is regulated by the same hormonal crosstalk network^21^ (Supplementary Methods), we have not studied the change in PIN1 patterning for *pin2* mutant.

**Figure 9:**
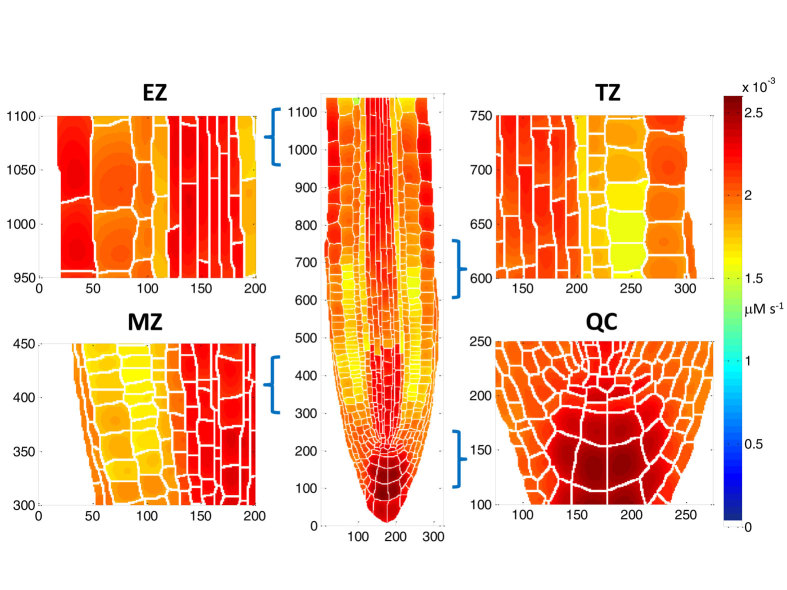
Modelling predictions for the patterning of auxin biosynthesis rate. EZ: elongation zone, TZ: transition zone, MZ: meristematic zone, QC: quiescent centre region.

Although the current study focuses on the potential relationships between auxin influx and efflux transporters for maintaining an auxin pattern, the model developed in this work is able to make predictions on the patterning of auxin biosynthesis rate. This is because auxin biosynthesis and degradation are also processes included in the hormonal crosstalk network (Supplementary Methods). Figure 9 summarises modelling predictions on the patterning of auxin biosynthesis rate. Specifically, auxin biosynthesis rates are increased towards the Arabidopsis root apex. In the QC and columella, auxin biosynthesis rates are high. In the epidermal cells of the elongation zone, auxin biosynthesis rates are also relatively high. These modelling predictions for auxin biosynthesis rate patterning are similar to those found by experimental observations (Fig. 5 in Petersson *et al*.^29^). We note that the modelled root in this study (Fig. 1) does not include the region for lateral root development. Therefore, modelling predictions can only be compared with the root in Fig. 5 in Petersson *et al*.^29^ after excluding the region of lateral root development. Moreover, the model also predicts that, in the transition zone, patterning of auxin biosynthesis rates is complex. Therefore, these modelling predictions reveal that elucidating the patterning of auxin biosynthesis needs to study the biosynthesis, degradation and transport of auxin as an integrative crosstalk system, as described in this work.

Since the model developed in this study has integrated auxin biosynthesis, degradation and transport, effects of any parameters relating to those processes can also be analysed using the model. For example, experimental measurement has shown that auxin apoplastic diffusion constant is smaller than that in water^30^. Figure S13 shows the effects on auxin patterning of reducing the auxin diffusion constant in the cell wall. Interestingly, modelling results predict that decreasing the auxin diffusion constant in cell wall favours auxin accumulation in the QC. In addition, the effects of varying levels of auxin influx and efflux carriers on auxin patterning can also be analysed. Figure S14 shows the effects of changing auxin influx levels on auxin patterning, and it supports the view that nonpolar AUX1/LAX carriers act to retain cellular auxin. Specifically, increasing/decreasing the levels of AUX1/LAX carriers affects the auxin concentration accordingly. These results reveal how nonpolar AUX1/LAX quantitatively contributes to the emergence of auxin patterning.

In summary, the model developed in this study is able not only to provide insights into the control of auxin patterning, but also to make various predictions that can be experimentally validated.

## Discussion

Experimental evidence shows that the quantitative properties of auxin gradients are important factors in regulating Arabidopsis root development. Auxin gradient formation is predominantly regulated by influx and efflux carriers that play distinct roles in controlling cellular auxin concentrations, by moving auxin into and out of the cell. However, PIN efflux proteins have polar localisation while AUX1/LAX influx proteins have nonpolar localisation in some cell types at least^27^. Since, for reasons given in this paper, the PIN3,4 and 7 auxin efflux carriers and the AUX1/LAX auxin influx carrier concentrations are prescribed and not established by the crosstalk network (Supplementary Methods), we cannot ‘predict’ or ‘explain’ how specific perturbations in one carrier set would affect the level and polarity of the other carrier set for PIN3,4,and 7 or AUX1/LAX. However, since the hormonal crosstalk network for PIN1,2 can be established^21^ (Supplementary Methods), effects of the prescribed auxin carriers on PIN1,2 patterning can be predicted and compared with experimental observations (Figure S12). While we cannot ‘predict’ all relationships between the influx and efflux carriers, the recovery principle does allow us to explore theoretically how the two carrier types could potentially coordinate their activity to establish and maintain auxin patterning and the possible implications for carrier concentration levels and polarity. In doing so, we effectively integrate the analysis of both carrier sets with auxin patterning rather than investigating the activity of one or other carrier type in isolation. This work is done within our existing crosstalk network, which includes auxin biosynthesis and degradation mechanisms.

By formulating a recovery principle, this work reveals how the level and localisation of both influx and efflux carriers are potentially interlinked to quantitatively control auxin pattern formation and how multiple combinations of levels and localisation of efflux and influx carriers could generate the same auxin pattern. Therefore, a specific auxin pattern is not uniquely determined by a specific efflux and influx carrier combination. The relationship between levels and localisation of these carriers plays the key role in determining a specific auxin pattern. When efflux carriers retain their original polarity but their levels are increased or decreased, both influx carrier level and polarity require simultaneous adjustment to maintain the original auxin pattern. Similarly, at different levels of influx carriers with the same nonpolar localisation, both the level and polarity of efflux carriers must also be simultaneously changed to maintain auxin patterning. Thus, the relationship between influx and efflux level and polarity, rather than the separate activity of either influx or efflux carrier, controls auxin pattern formation.

The roles of influx and efflux carriers in plant development have been subjected to extensive investigation, and existing research predominantly explains experimental observations by the activity of auxin influx carriers^14^,^27^,^31^,^32^,^33^,^34^,^35^ the activity of efflux carriers^7^ (references therein), or the ABCB transporters^9^,^10^,^36^,^37^. The model developed in this work has integrated the information for all of these important known auxin transporters rather than treating them as separate independent entities.

Although accumulated experimental evidence demonstrates that both auxin influx and efflux carriers have roles in plant development, a major obstacle for elucidating auxin patterning is the lack of a methodology to integrate the functions of these carriers. The recovery principle describes a methodology which allows the quantitative integrated analysis of the linkage between auxin and influx and efflux carrier patterning (Fig. 8a). As we have demonstrated, this reveals how levels and localisation of influx and efflux carriers can coordinate their activity to maintain a specific auxin pattern. This enables searchable relationships between influx and efflux activity for a target auxin pattern (Fig. 8b), sheds light on the integrated actions of influx and efflux carriers, and suggests that further understanding of the roles of auxin carriers in auxin patterning requires the study of the relationships between the carriers, as well as the study of each individual carrier. Previous modelling analysis has made efforts to study the actions of both influx and efflux carriers, but tends to emphasize the independent activities of either the efflux^12^ or the influx carriers^13^.

This work also demonstrates that once auxin patterning is recovered using the recovery principle, PIN1 and PIN2 patterning also recovers due to the action of hormonal crosstalk, which suggests that auxin controls the patterning of its own transporters via hormonal crosstalk. Control of patterning of the other transporters can be elucidated in the future, by conducting further experimental research and combining experimental data with modelling analysis. The design of specific experimental measurements will be critical to provide the necessary data for constructing hormonal crosstalk networks for these transporters.

Mechanisms of polar auxin transport have been subjected to extensive study using mathematical models. A recent excellent review has discussed various models and mechanisms^38^. These can be divided into flux-based or concentration-based models which can be used to study polar auxin transport mechanisms^38^. However they only consider the relationships between auxin and PIN efflux carriers. This work reveals that the relationships between influx and efflux level and polarity (rather than separate influx or efflux carrier activity) could control auxin pattern formation. A recent modelling study has suggested that auxin influx carriers can play an important role in polarising PIN carriers^39^. Future research on possible mechanisms for polar auxin transport should therefore study the combined roles of efflux and influx carriers, focusing on how the relationship between auxin and polar PIN depends on the levels and localisation of the influx carriers.

Whilst this research has integrated a wide range of experimental data to establish a data-driven mechanistic model for elucidating the control of auxin patterning and for making various predictions, we only consider the current model be a starting point. Clearly, our model can only analyse the contribution to auxin patterning of those components in the hormonal crosstalk network (Supplementary Methods). Future challenges include the integration of other hormones, signalling molecules and some relevant gene expression processes into the model; and combination of further modelling and experimental studies to elucidate the contribution of each process to auxin patterning. These future efforts should be able to further elucidate the relationships of auxin patterning, polarity and patterning of multiple efflux carriers, multiple influx carriers patterning, and auxin biosynthesis, degradation and conjugation.

## Methods

### Root structure with realistic cell geometries, polar localisation of efflux carriers and nonpolar localisation of influx carriers

A root structure with realistic cell geometries was constructed using experimental images^13^. Each cell consists of a cytosolic space contained by a plasma membrane, surrounded by its own cell wall. The area between adjacent cell walls represents extracellular space. Auxin diffuses within the cytosol, or within the combined cell wall and extra-cellular space, while transport between the cytosol and cell wall is facilitated by influx and efflux carriers. Details for constructing a realistic root structure that integrates actual cell geometries, the level and polar or nonpolar localisation of auxin influx and efflux carriers, with a variety of experimental data about hormonal crosstalk, are included in the Supplementary Methods.

### Hormonal crosstalk

Crosstalk network between auxin, ethylene, cytokinin and PIN1 and PIN2 is included in Supplementary Methods. It was previously constructed by iteratively combining modelling and experimental measurements^21^,^22^,^23^. Extensive examination of published experimental data reveals that it is currently not possible to construct a network between the three hormones and other carriers, due to insufficient data and the complexity of crosstalk between hormones and auxin carriers, as discussed in Supplementary Methods. Therefore, PIN3,4,7, AUX1 and LAX2 and 3 localisation is prescribed using experimental data, and the details are included in Supplementary Methods.

### Numerical methods

The partial differential equations, describing spatiotemporal dynamics of hormonal crosstalk in the root with multiple influx and efflux carriers, is solved using the finite volume method, in which each gridpoint is used as an element to establish the discrete mass balance equations. The numerical method was previously described in detail^21^.

## Additional Information

**How to cite this article:** Moore, S. *et al*. A recovery principle provides insight into auxin pattern control in the Arabidopsis root. *Sci. Rep.* **7**, 43004; doi: 10.1038/srep43004 (2017).

**Publisher's note:** Springer Nature remains neutral with regard to jurisdictional claims in published maps and institutional affiliations.

## Supplementary Material

Supplementary Figures and Methods

## References

[b1] Vanneste S. & Friml J. Auxin: A Trigger for Change in Plant Development. Cell 136, 1005–1016 (2009). 19303845 10.1016/j.cell.2009.03.001

[b2] Sabatini S. . An auxin-dependent distal organizer of pattern and polarity in the Arabidopsis root. Cell 99, 463–472 (1999). 10589675 10.1016/s0092-8674(00)81535-4

[b3] Dubrovsky J. G. . Auxin minimum defines a developmental window for lateral root initiation. New Phytol. 191, 970–983 (2011). 21569034 10.1111/j.1469-8137.2011.03757.x

[b4] Peret B. . Sequential induction of auxin efflux and influx carriers regulates lateral root emergence. Mol. Syst. Biol. 9, 699 (2013). 24150423 10.1038/msb.2013.43 PMC3817398

[b5] Moubayidin L. . The rate of cell differentiation controls the Arabidopsis root meristem growth phase. Curr. Biol. 20, 1138–43 (2010). 20605455 10.1016/j.cub.2010.05.035

[b6] Zazimalova E., Murphy A. S., Yang H., Hoyerova K. & Hosek P. Auxin transporters—why so many? Cold Spring Harbor Persp. Biol. 2, a001552 (2010). 10.1101/cshperspect.a001552 PMC2829953 20300209

[b7] Adamowski M. & Friml J. PIN-dependent auxin transport: action, regulation, and evolution. Plant Cell 27, 20–32 (2015). 25604445 10.1105/tpc.114.134874 PMC4330589

[b8] Swarup R. & Peret B. AUX/LAX family of auxin influx carriers-an overview. Front. Plant Sci. 3, 225 (2012). 23087694 10.3389/fpls.2012.00225 PMC3475149

[b9] Geisler M. & Murphy A. S. The ABC of auxin transport: The role of p-glycoproteins in plant development. FEBS Lett. 580, 1094–1102 (2006). 16359667 10.1016/j.febslet.2005.11.054

[b10] Cho M. & Cho H. T. The function of ABCB transporters in auxin transport. Plant Signal. Behav. 8, e22990 (2012). 23221777 10.4161/psb.22990 PMC3656995

[b11] Friml J. . AtPIN4 mediates sink-driven auxin gradients and root patterning in Arabidopsis. Cell 108, 661–673 (2002). 11893337 10.1016/s0092-8674(02)00656-6

[b12] Grieneisen V. A., Xu J., Maree A. F. M., Hogeweg P. & Scheres B. Auxin transport is sufficient to generate a maximum and gradient guiding root growth. Nature 449, 1008–1013 (2007). 17960234 10.1038/nature06215

[b13] Band L. R. . Systems analysis of auxin transport in the Arabidopsis root apex. Plant Cell 26, 862–875 (2014). 24632533 10.1105/tpc.113.119495 PMC4001398

[b14] Swarup R. . Root gravitropism requires lateral root cap and epidermal cells for transport and response to a mobile auxin signal. Nat. Cell Biol. 7, 1057–1065 (2005). 16244669 10.1038/ncb1316

[b15] De Rybel B. . Plant development. Integration of growth and patterning during vascular tissue formation in Arabidopsis. Science 345, 1255215 (2014). 25104393 10.1126/science.1255215

[b16] Xuan W. . Cyclic programmed cell death stimulates hormone signalling and root development in Arabidopsis. Science 351, 384–38 (2016). 26798015 10.1126/science.aad2776

[b17] Müller A. . AtPIN2 defines a locus of Arabidopsis for root gravitropism control. EMBO J. 17, 6903–6911 (1998). 9843496 10.1093/emboj/17.23.6903 PMC1171038

[b18] Blilou I. . The PIN auxin efflux facilitator network controls growth and patterning in Arabidopsis roots. Nature 433, 39–44 (2005). 15635403 10.1038/nature03184

[b19] Kleine-Vehn J. . Cellular and molecular requirements for polar PIN targeting and transcytosis in plants. Mol. Plant 1, 1056–1066 (2008). 19825603 10.1093/mp/ssn062

[b20] Laskowski M. . Root system architecture from coupling cell shape to auxin transport. PLoS Biol. 6 (2008). 10.1371/journal.pbio.0060307 PMC2602721 19090618

[b21] Moore S. . Spatiotemporal modelling of hormonal crosstalk explains the level and patterning of hormones and gene expression in Arabidopsis thaliana wildtype and mutant roots. New Phytol. 207, 1110–1122 (2015). 25906686 10.1111/nph.13421 PMC4539600

[b22] Liu J. L., Mehdi S., Topping J., Tarkowski P. & Lindsey K. Modelling and experimental analysis of hormonal crosstalk in Arabidopsis. Molec. Syst. Biol. 6, 373 (2010). 20531403 10.1038/msb.2010.26 PMC2913391

[b23] Liu J. L., Mehdi S., Topping J., Friml J. & Lindsey K. Interaction of PLS and PIN and hormonal crosstalk in Arabidopsis root development. Front. Plant Sci. 4, 75 (2013). 23577016 10.3389/fpls.2013.00075 PMC3617403

[b24] Liao C.-Y. . Reporters for sensitive and quantitative measurement of auxin response. Nature Meth. 12, 207–210 (2015). 10.1038/nmeth.3279 PMC4344836 25643149

[b25] Bishopp A. . A mutually inhibitory interaction between auxin and cytokinin specifies vascular pattern in roots. Curr. Biol. 21, 917–926 (2011). 21620702 10.1016/j.cub.2011.04.017

[b26] Rowe J. H., Topping J. F., Liu J. & Lindsey K. Abscisic acid regulates root growth under osmotic stress conditions via an interacting hormonal network with cytokinin, ethylene and auxin. New Phytol. 211, 225–239 (2016). 26889752 10.1111/nph.13882 PMC4982081

[b27] Peret B. . AUX/LAX genes encode a family of auxin influx transporters that perform distinct functions during Arabidopsis development. Plant Cell 24, 2874–2885 (2012). 22773749 10.1105/tpc.112.097766 PMC3426120

[b28] Omelyanchuk N. A. . A detailed expression map of the PIN1 auxin transporter in *Arabidopsis thaliana* root. BMC Plant Biol. 16 (Suppl 1), 5 (2016). 26821586 10.1186/s12870-015-0685-0 PMC4895256

[b29] Petersson S. V. . An auxin gradient and maximum in the Arabidopsis root apex shown by high-resolution cell-specific analysis of IAA distribution and synthesis. Plant Cell 21, 1659–1668 (2009). 19491238 10.1105/tpc.109.066480 PMC2714926

[b30] Kramer E. M., Frazer N. L. & Baskin T. I. Measurement of diffusion within the cell wall in living roots of *Arabidopsis thaliana*. J. Exp. Bot. 58, 3005–3015 (2007). 17728296 10.1093/jxb/erm155

[b31] Swarup R. . Localization of the auxin permease AUX1 suggests two functionally distinct hormone transport pathways operate in the Arabidopsis root apex. Genes Dev. 15, 2648–2653 (2001). 11641271 10.1101/gad.210501 PMC312818

[b32] Dharmasiri S. . AXR4 is required for localization of the auxin influx facilitator AUX1. Science 312, 1218–1220 (2006). 16690816 10.1126/science.1122847

[b33] Ugartechea-Chirino Y.. The AUX1 LAX family of auxin influx carriers is required for the establishment of embryonic root cell organization in Arabidopsis thaliana. Ann. Bot. 105, 277–289 (2010). 19952011 10.1093/aob/mcp287 PMC2814760

[b34] Robert H. S. . Plant embryogenesis requires AUX/LAX-mediated auxin influx. Development 142, 702–11 (2015). 25617434 10.1242/dev.115832

[b35] Fàbregas N. . Auxin influx carriers control vascular patterning and xylem differentiation in Arabidopsis thaliana. PLoS Genet. 11, e1005183 (2015). 25922946 10.1371/journal.pgen.1005183 PMC4414528

[b36] Geisler M. . Cellular efflux of auxin catalyzed by the Arabidopsis MDR/PGP transporter AtPGP1. Plant J. 44, 179–194 (2005). 16212599 10.1111/j.1365-313X.2005.02519.x

[b37] Cho M., Lee S. H. & Cho H. T. P-glycoprotein4 displays auxin efflux transporter-like action in Arabidopsis root hair cells and tobacco cells. Plant Cell 19, 3930–3943 (2007). 18156217 10.1105/tpc.107.054288 PMC2217643

[b38] van Berkel K., de Boer R. J., Scheres B. & ten Tusscher K. Polar auxin transport: models and mechanisms. Development 140, 2253–2268 (2013). 23674599 10.1242/dev.079111

[b39] Cieslak M., Runions A. & Prusinkiewicz P. Auxin-driven patterning with unidirectional fluxes. J. Exp. Bot. 66, 5085–5102 (2015). 10.1093/jxb/erv262 PMC4513925 26116915

