# Supplementary material for: A recovery principle provides insight into auxin pattern control in the Arabidopsis root
Source: Sci Rep. 2017 Feb 21;7:43004. doi: 10.1038/srep43004 (PMC5318957; doi:10.1038/srep43004)
Supplement: Supplementary Figures and Methods [file srep43004-s1.pdf]

Supplementary Material:

A recovery principle provides insight into auxin pattern control in the Arabidopsis root

Simon Moore[1†], Junli Liu[1†*], Xiaoxian Zhang[2] and Keith Lindsey[1*]

[1]Department of Biosciences, Durham University, South Road, Durham DH1 3LE, UK

[2] Department of Sustainable Soil and Grassland System, Rothamsted Research, Harpenden, Hertfordshire AL5 2GQ, UK

[*†]Joint first authors: both authors contributed equally to this work.

[*]Joint corresponding authors

Authors for correspondence:

Keith Lindsey (keith.lindsey@durham.ac.uk, tel: +44 191 334 1309; fax: +44 191 334 1201).

Junli Liu (Junli.Liu@durham.ac.uk, tel: +44 191 3341376)

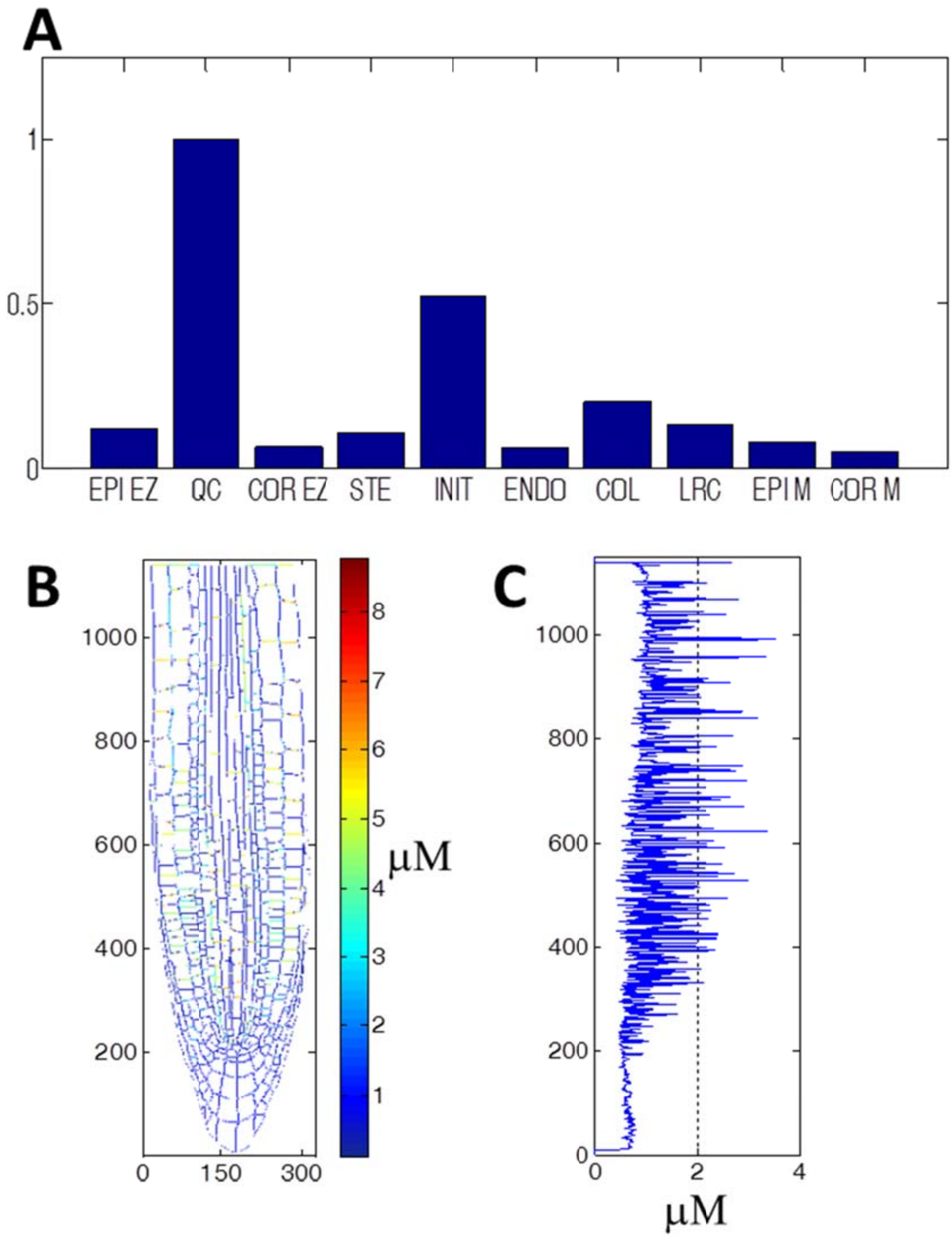

Figure S1. Wildtype auxin and PIN1,2 patterning. (**A**) Auxin concentrations in different cell types relative to the QC, which is set to unity. (EZ: elongation zone; M: meristematic zone; EPI: epidermis; QC: quiescent centre; COR: cortex; STE: stele; INIT: initials; ENDO: endodermis; COL: columella; LRC: lateral root cap). (**B**) Concentration colour map of both PIN1 and PIN2 at the plasma membrane. (**C**) Concentration profile for both PIN1 and PIN2 at the plasma membrane, showing the trend in average cross-sectional concentration along the root tip.

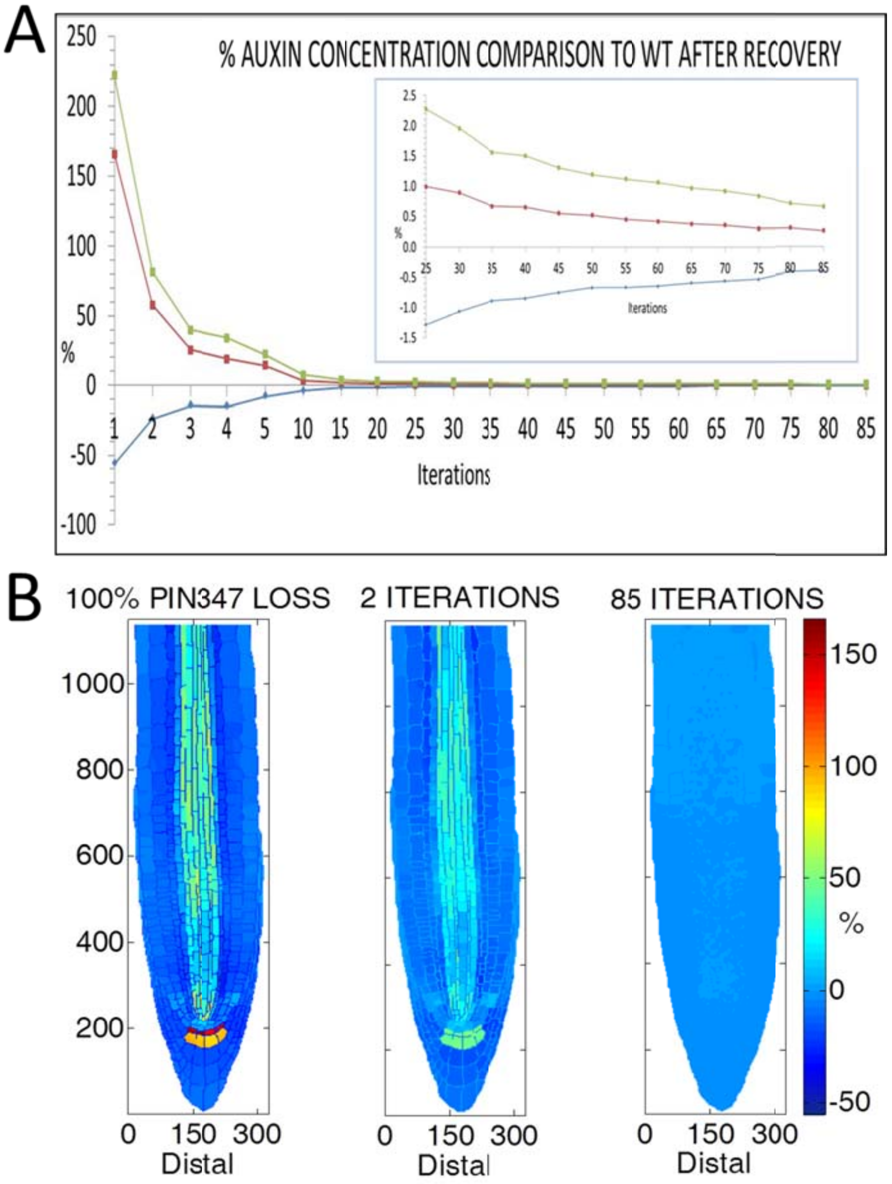

Figure S2. Auxin pattern recovery after 100% loss in total wildtype PIN3,4,7 concentrations, using the recovery principle over 85 iterations. **(A)** Maximum percentage difference of auxin concentration from wildtype. Blue curve shows maximum percentage difference of auxin concentration below wildtype for all data points in the root at each iteration. Red curve shows maximum percentage difference of auxin concentration above wildtype for all data points in the root at each iteration. Green curve shows maximum percentage difference range from wildtype within the root and it is calculated by adding the absolute values of the data in the blue and red curves at each iteration. **(B)** Colour map images of the percentage difference from wildtype auxin concentrations in the root after the initial perturbation of 100% loss of PIN3,4,7, then after 2 recovery iterations, and full recovery after 85 iterations.

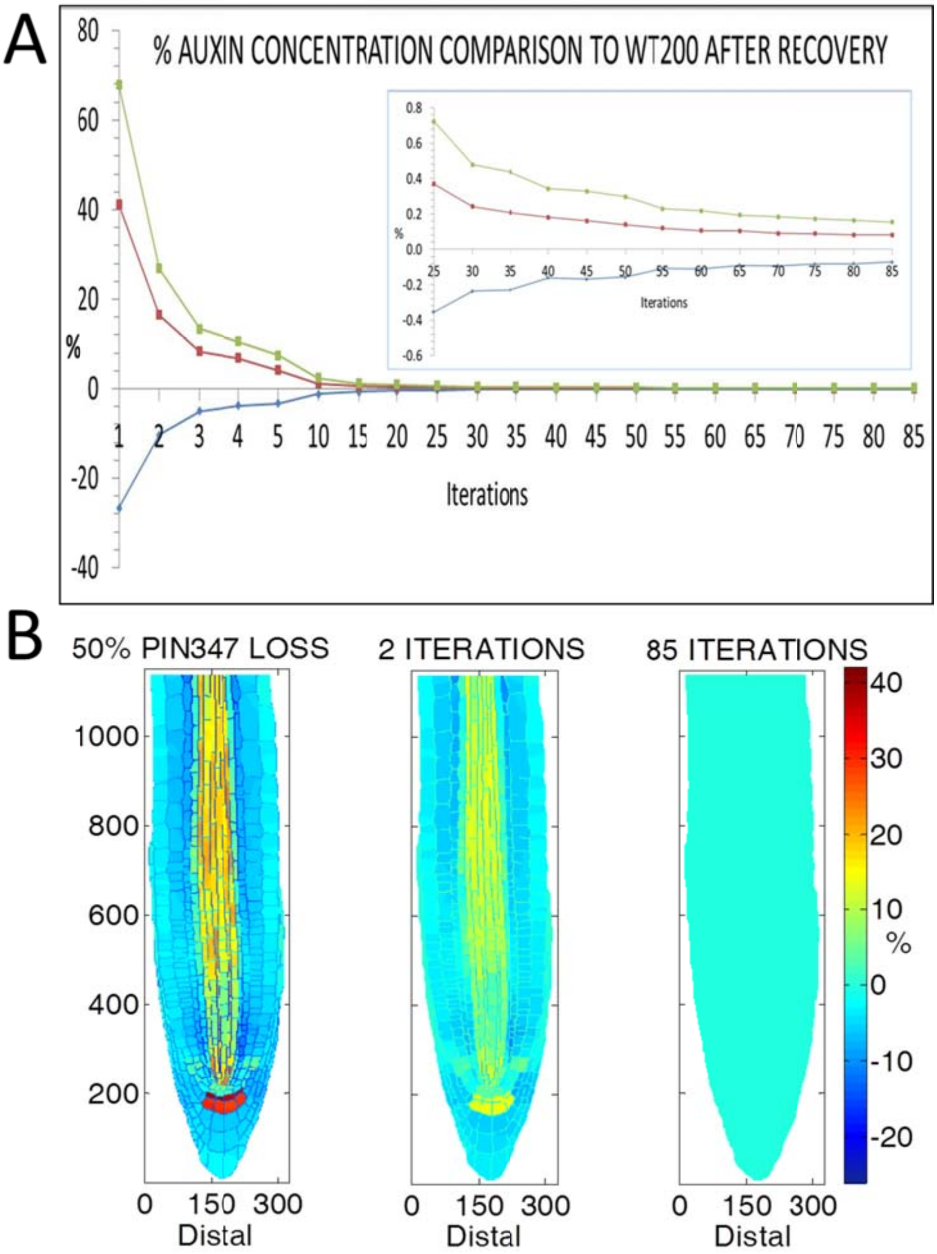

Figure S3. Auxin pattern recovery after 50% loss in total wildtype PIN3,4,7 concentrations, using the recovery principle over 85 iterations. **(A)** Maximum percentage difference of auxin concentration from wildtype. Blue curve shows maximum percentage difference of auxin concentration below wildtype for all data points in the root at each iteration. Red curve shows maximum percentage difference of auxin concentration above wildtype for all data points in the root at each iteration. Green curve shows maximum percentage difference range from wildtype within the root and it is calculated by adding the absolute values of the data in the blue and red curves at each iteration. **(B)** Colour map images of the percentage difference from wildtype auxin concentrations in the root after the initial perturbation of 50% loss of PIN3,4,7, then after 2 recovery iterations, and full recovery after 85 iterations.

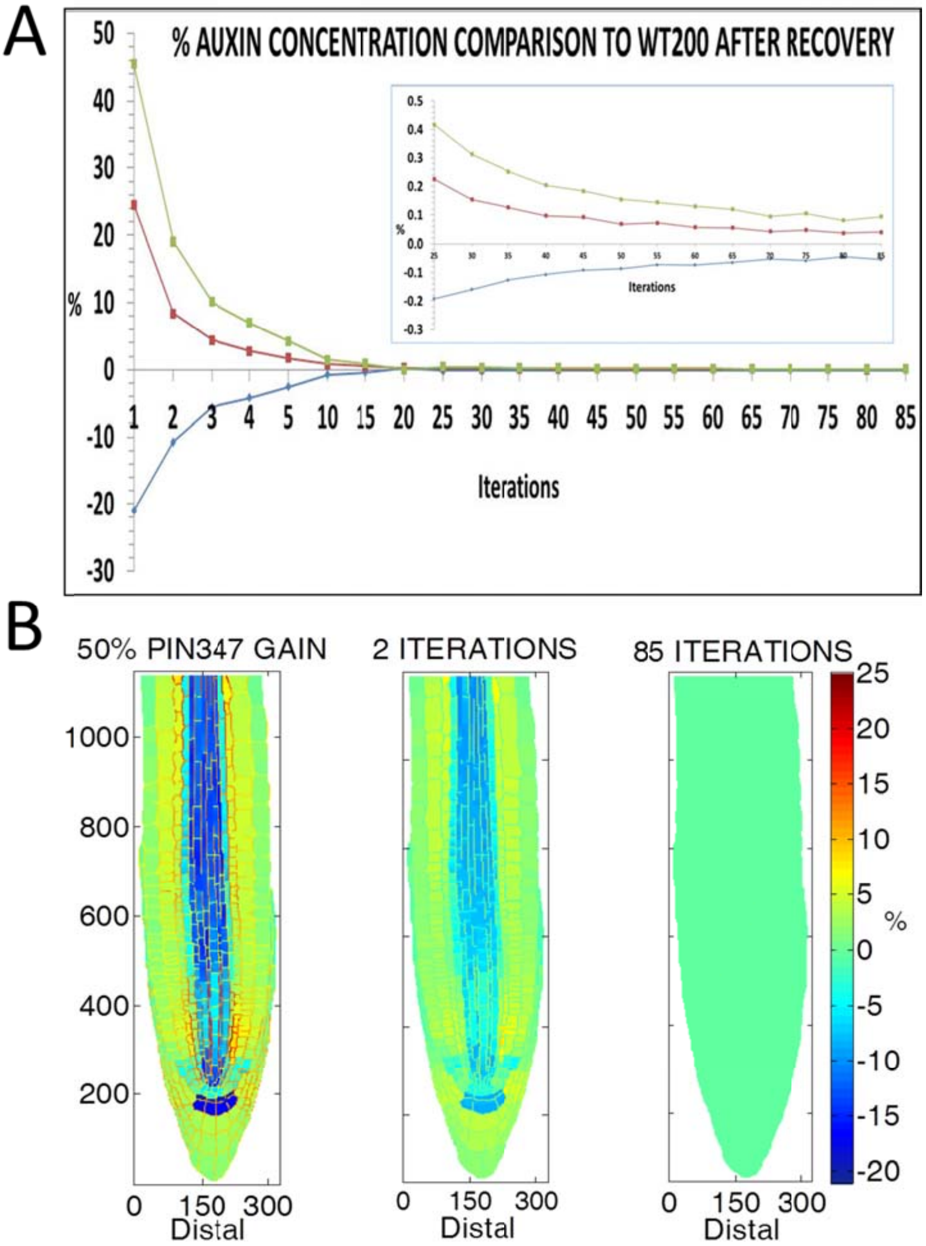

Figure S4. Auxin pattern recovery after 50% gain in total wildtype PIN3,4,7 concentrations, using the recovery principle over 85 iterations. **(A)** Maximum percentage difference of auxin concentration from wildtype. Blue curve shows maximum percentage difference of auxin concentration below wildtype for all data points in the root at each iteration. Red curve shows maximum percentage difference of auxin concentration above wildtype for all data points in the root at each iteration. Green curve shows maximum percentage difference range from wildtype within the root and it is calculated by adding the absolute values of the data in the blue and red curves at each iteration. **(B)** Colour map images of the percentage difference from wildtype auxin concentrations in the root after the initial perturbation of 50% gain of PIN3,4,7, then after 2 recovery iterations, and full recovery after 85 iterations.

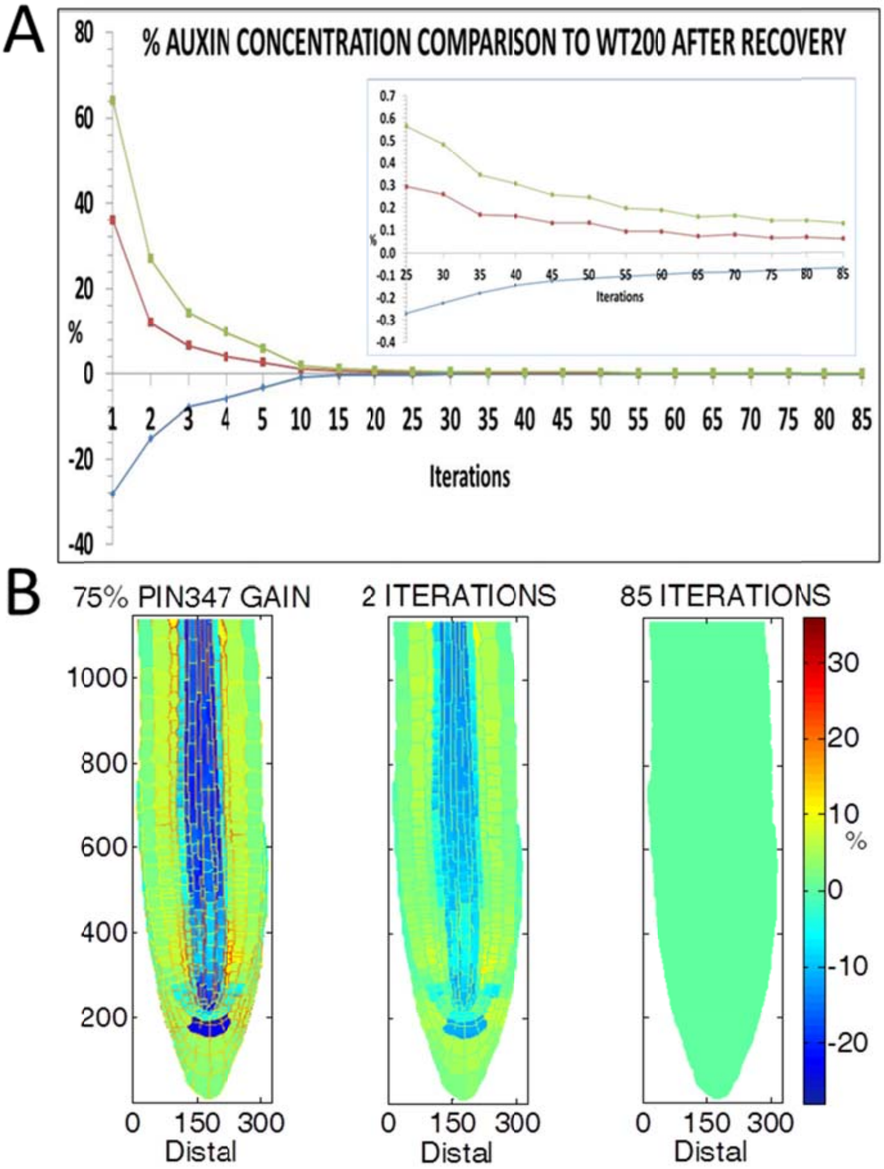

Figure S5. Auxin pattern recovery after 75% gain in total wildtype PIN3,4,7 concentrations, using the recovery principle over 85 iterations. **(A)** Maximum percentage difference of auxin concentration from wildtype. Blue curve shows maximum percentage difference of auxin concentration below wildtype for all data points in the root at each iteration. Red curve shows maximum percentage difference of auxin concentration above wildtype for all data points in the root at each iteration. Green curve shows maximum percentage difference range from wildtype within the root and it is calculated by adding the absolute values of the data in the blue and red curves at each iteration. **(B)** Colour map images of the percentage difference from wildtype auxin concentrations in the root after the initial perturbation of 75% gain of PIN3,4,7, then after 2 recovery iterations, and full recovery after 85 iterations.

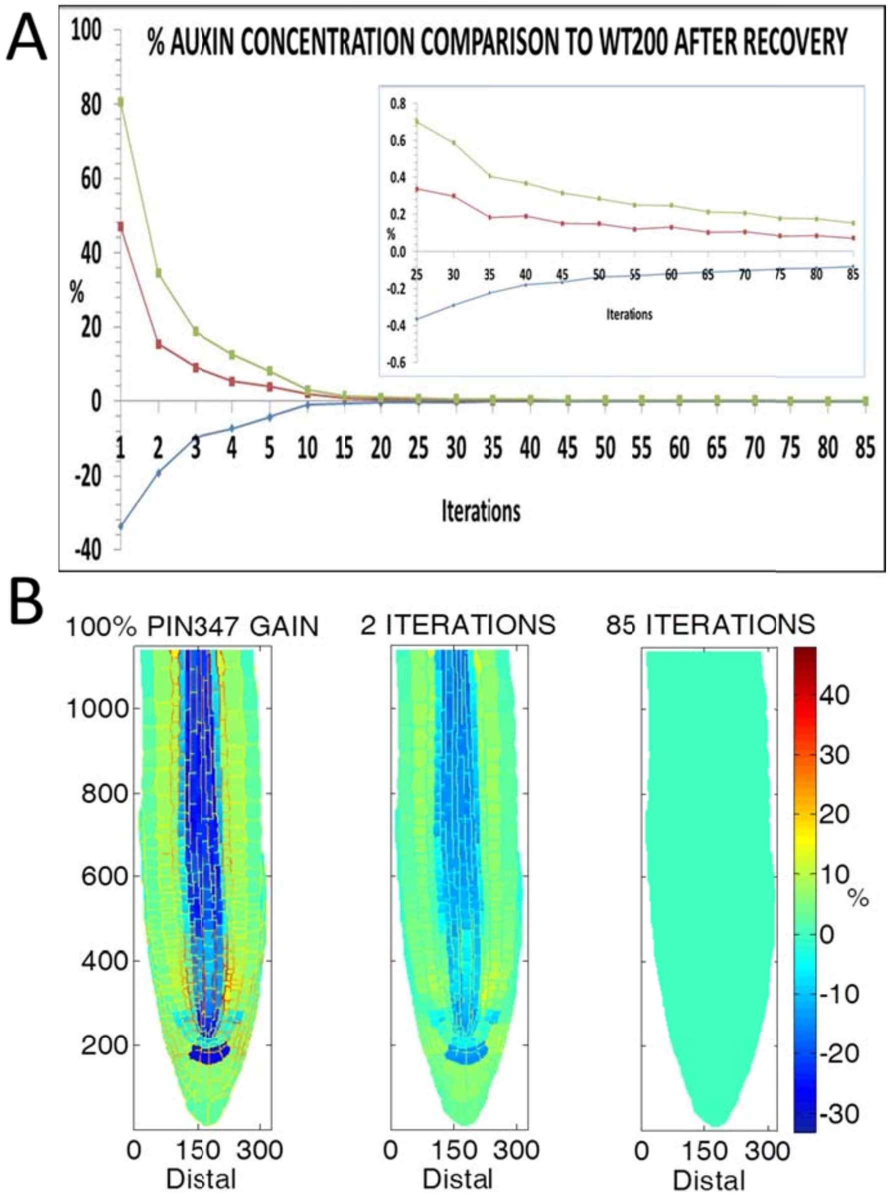

Figure S6. Auxin pattern recovery after 100% gain in total wildtype PIN3,4,7 concentrations, using the recovery principle over 85 iterations. **(A)** Maximum percentage difference of auxin concentration from wildtype. Blue curve shows maximum percentage difference of auxin concentration below wildtype for all data points in the root at each iteration. Red curve shows maximum percentage difference of auxin concentration above wildtype for all data points in the root at each iteration. Green curve shows maximum percentage difference range from wildtype within the root and it is calculated by adding the absolute values of the data in the blue and red curves at each iteration. **(B)** Colour map images of the percentage difference from wildtype auxin concentrations in the root after the initial perturbation of 100% gain of PIN3,4,7, then after 2 recovery iterations, and full recovery after 85 iterations.

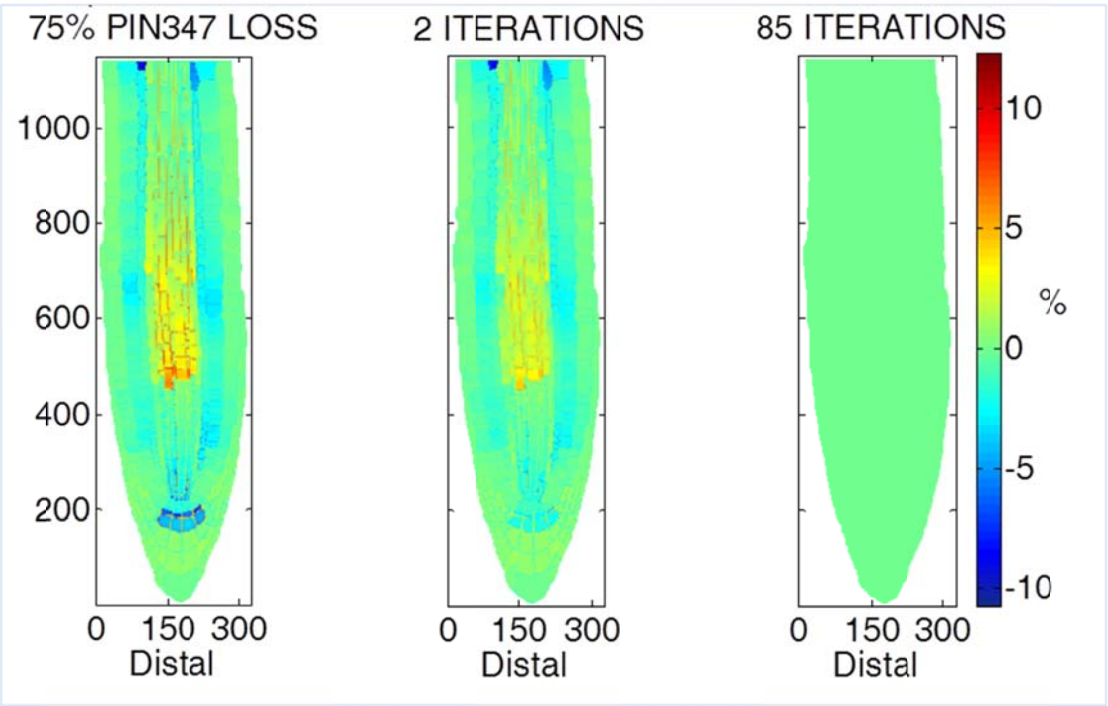

Figure S7. PIN1 and PIN2 recovery after 75% decrease in total wildtype PIN3,4,7 concentrations, using the recovery principle over 85 iterations. Colour map images show the percentage difference from wildtype concentration of PIN1 and PIN2 in the root after the initial perturbation of 75% loss of PIN3,4,7, then after 2 recovery iterations, and full recovery after 85 iterations.

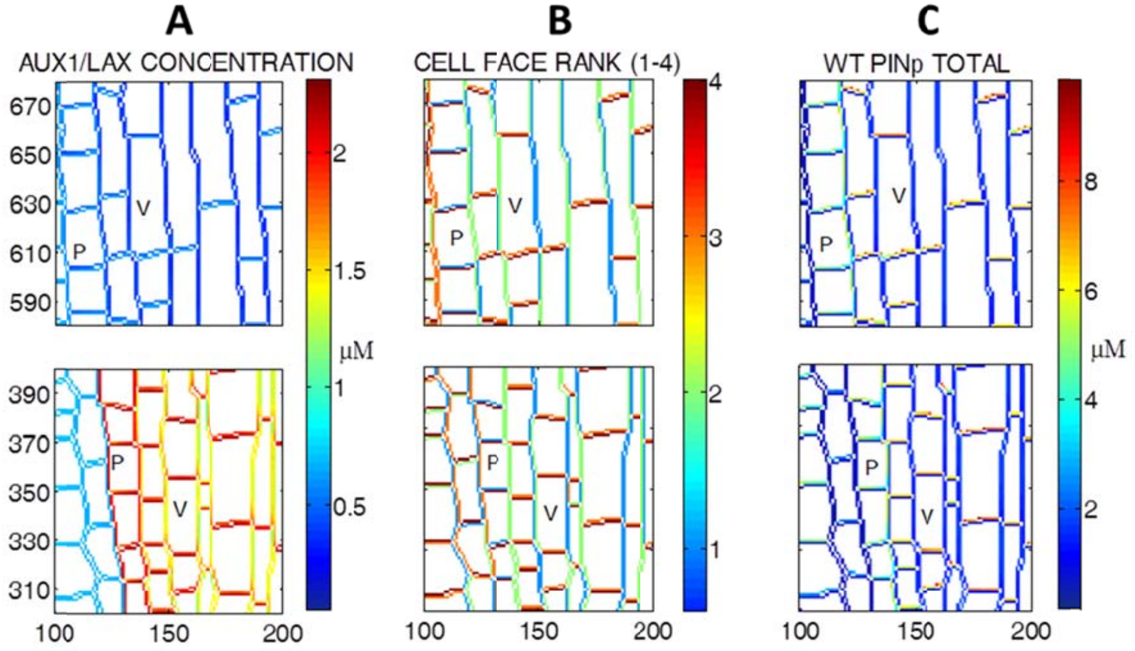

Figure S8. Two enlarged regions of the root, showing the AUX1/LAX concentration patterning required for auxin pattern recovery from 75% loss in wildtype total PIN347. (**A**) AUX1/LAX colour map for recovery requires non-uniform and polar distribution of AUX1/LAX. (**B**) AUX1/LAX average cell face concentrations are ranked 1 to 4 for each cell. This is calculated by averaging the data in each cell face in (A) and by ranking them in terms of the averaged values. Imaging of the concentration rankings demonstrates polar AUX1/LAX distribution. (**C**) Polar distribution of total PIN proteins (summation of PIN1,2,3,4,7) in wildtype (P: pericycle cell, V: vascular cell).

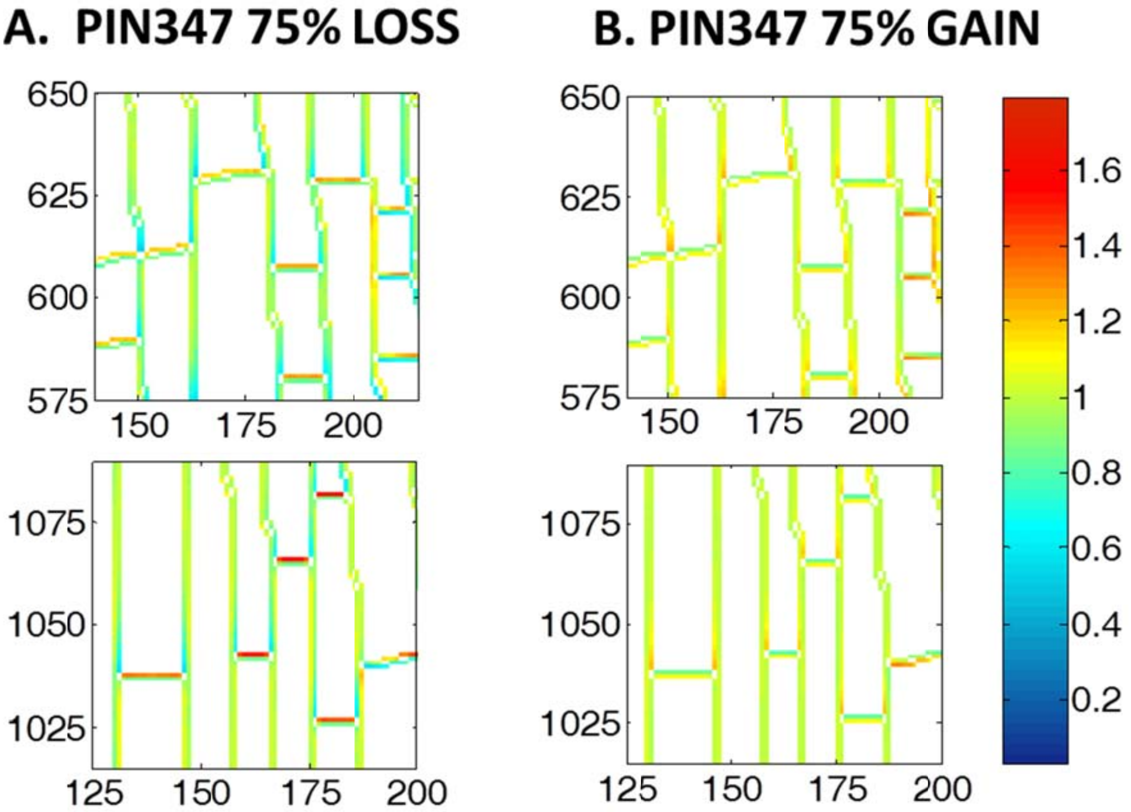

Figure S9. PIN and AUX1/LAX patterns that maintain the same auxin pattern do not exhibit spatially proportional correlation. This figure is the ratio of two ratios: ratio1/ratio2. ratio1: **(TOTAL PIN AFTER RECOVERY) /(TOTAL PIN IN WILDTYPE);** ratio2: (**TOTAL AUX1LAX AFTER RECOVERY) /(TOTAL AUX1LAX IN WILDTYPE).** (A): Recovery from 75% PIN347 loss. (B): Recovery from 75% PIN347 gain. Both (A) and (B) show the same two regions of the root, and they demonstrate that, although auxin patterning is recovered for both cases, this ratio of ratios is generally not unity. This result implies that PIN and AUX1/LAX patterns that maintain the same auxin pattern do not exhibit spatially proportional correlation. Comparing figures A and B shows that this ratio of ratios is different for 75% loss of PIN3,4,7 from that for 75% gain of PIN3,4,7. This result implies that 75% loss of PIN3,4,7 and 75% gain of PIN3,4,7 require different relationships between PIN and AUX1/LAX to maintain the same auxin patterning.

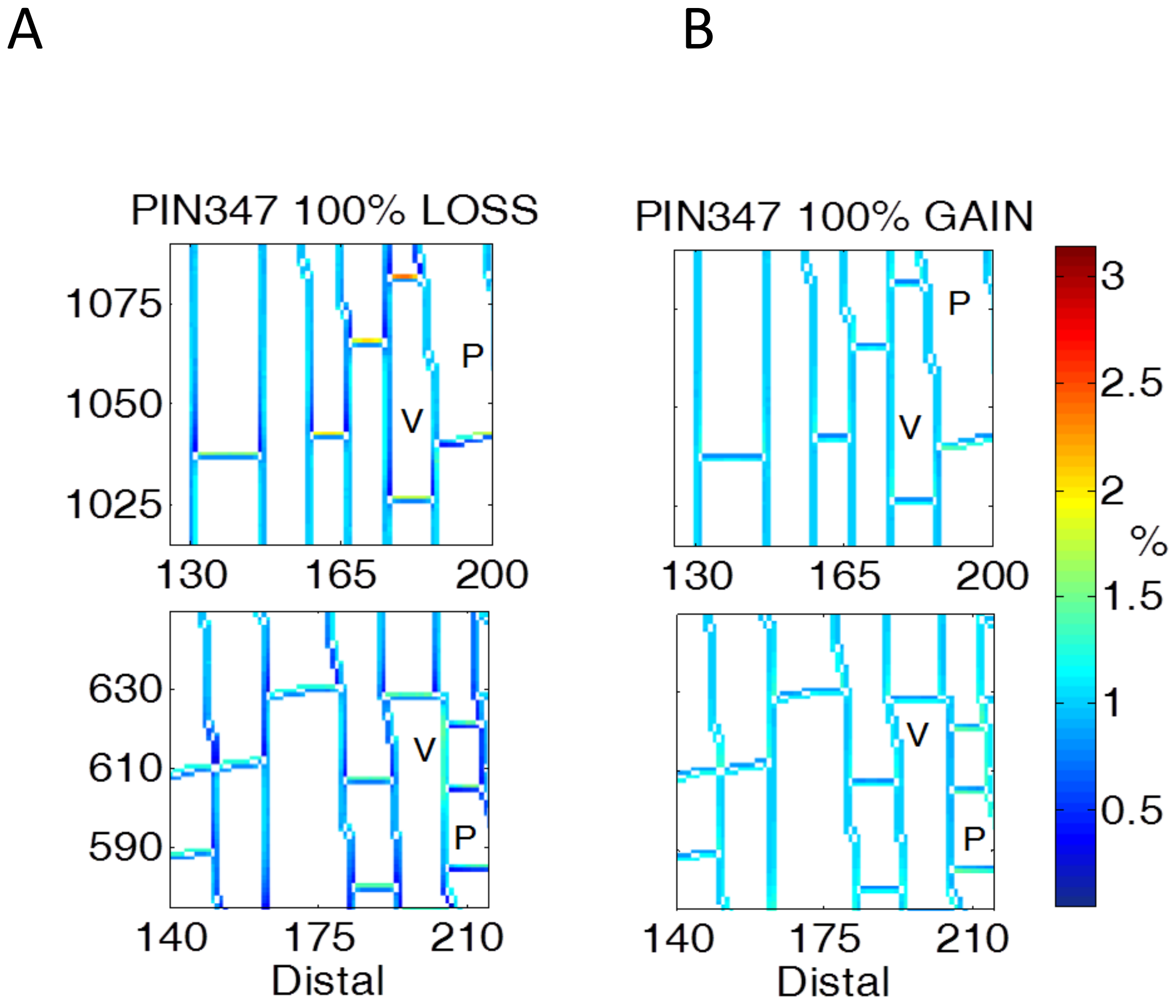

Figure S10. PIN and AUX1/LAX patterns that maintain the same auxin pattern do not exhibit spatially proportional correlation. This figure is the ratio of two ratios: ratio1/ratio2. ratio1: **(TOTAL PIN AFTER RECOVERY) /(TOTAL PIN IN WILDTYPE);** ratio2: **(TOTAL AUX1LAX AFTER RECOVERY) /(TOTAL AUX1LAX IN WILDTYPE).** (a): Recovery from 100% PIN347 loss. (b): Recovery from 100% PIN347 gain. Both (a) and (b) show the same two regions of the root, and they demonstrate that, although auxin patterning is recovered for both cases, this ratio of ratios is generally not unity. This result implies that PIN and AUX1/LAX patterns that maintain the same auxin pattern do not exhibit spatially proportional correlation. Comparing figures A and B shows that this ratio of ratios is different for 100% loss of PIN3,4,7 from that for 100% gain of PIN3,4,7. This result implies that 100% loss of PIN3,4,7 and 100% gain of PIN3,4,7 require different relationships between PIN and AUX1/LAX to maintain the same auxin patterning.

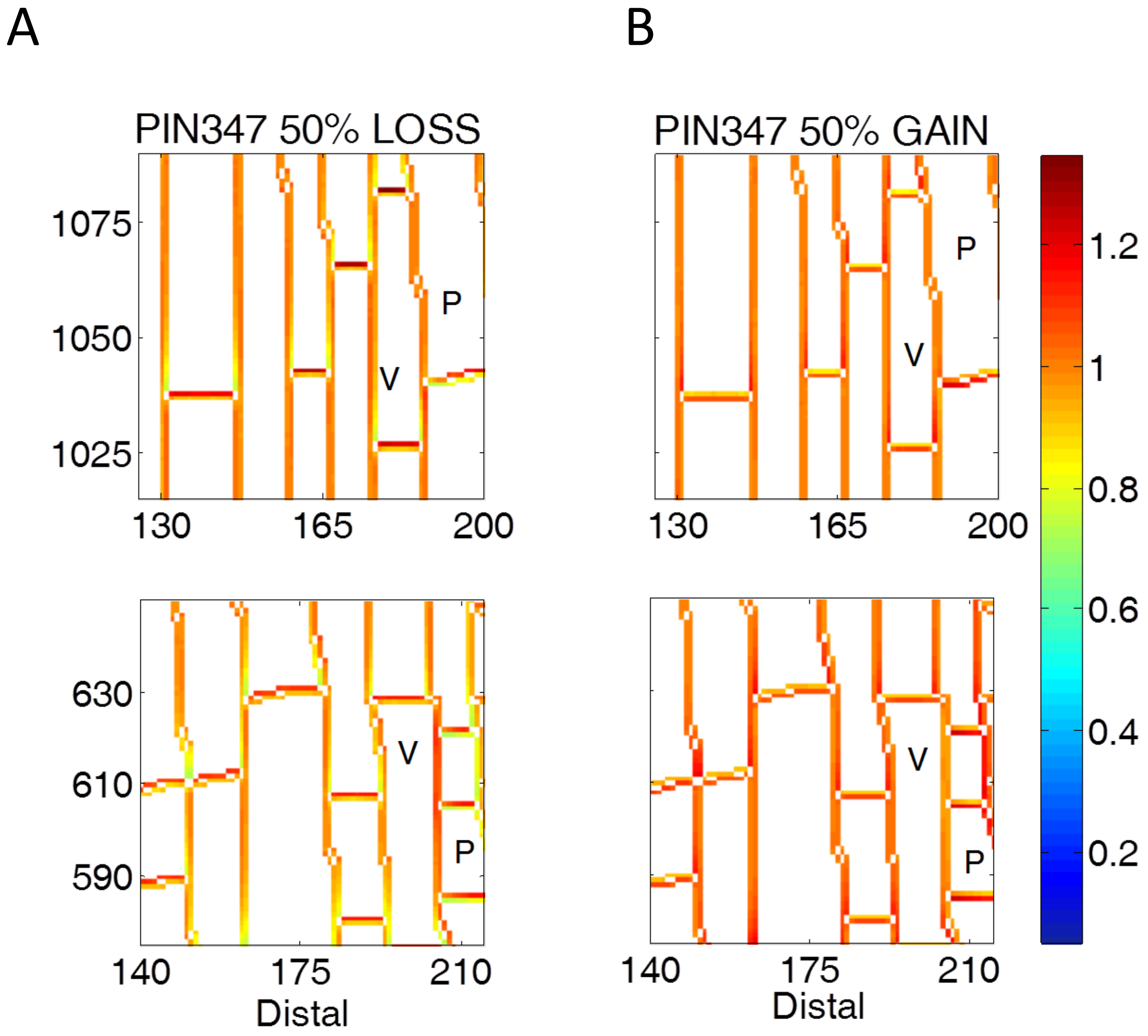

Figure S11. PIN and AUX1/LAX patterns that maintain the same auxin pattern do not exhibit spatially proportional correlation. This figure is the ratio of two ratios: ratio1/ratio2. ratio1: **(TOTAL PIN AFTER RECOVERY) /(TOTAL PIN IN WILDTYPE);** ratio2: **(TOTAL AUX1LAX AFTER RECOVERY) /(TOTAL AUX1LAX IN WILDTYPE).** (a): Recovery from 50% PIN347 loss. (b): Recovery from 50% PIN347 gain. Both (A) and (B) show the same two regions of the root, and they demonstrate that, although auxin patterning is recovered for both cases, this ratio of ratios is generally not unity. This result implies that PIN and AUX1/LAX patterns that maintain the same auxin pattern do not exhibit spatially proportional correlation. Comparing figures A and B shows that this ratio of ratios is different for 50% loss of PIN3,4,7 from that for 50% gain of PIN3,4,7. This result implies that 50% loss of PIN3,4,7 and 50% gain of PIN3,4,7 require different relationships between PIN and AUX1/LAX to maintain the same auxin patterning.

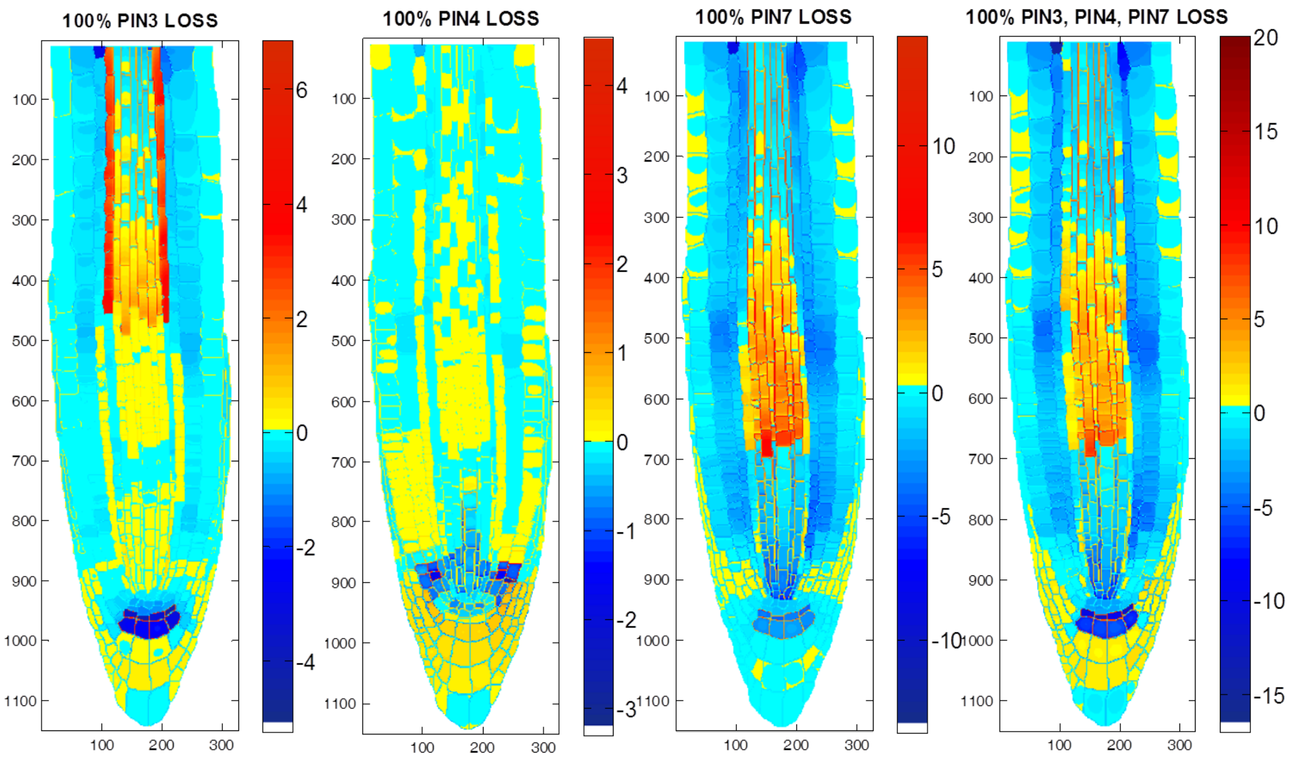

Figure S12. Modelling predictions on the combined PIN1 and PIN2 concentration patterns for 100% loss of PIN3 or PIN4 or PIN7 and for the combined 100% loss of PIN3, PIN4 and PIN7. This figure shows percentage difference of PIN1,2 concentration from wildtype. From left to right, PIN3, PIN4, PIN7 or total PIN3,4,7 concentration is set to be zero, respectively.

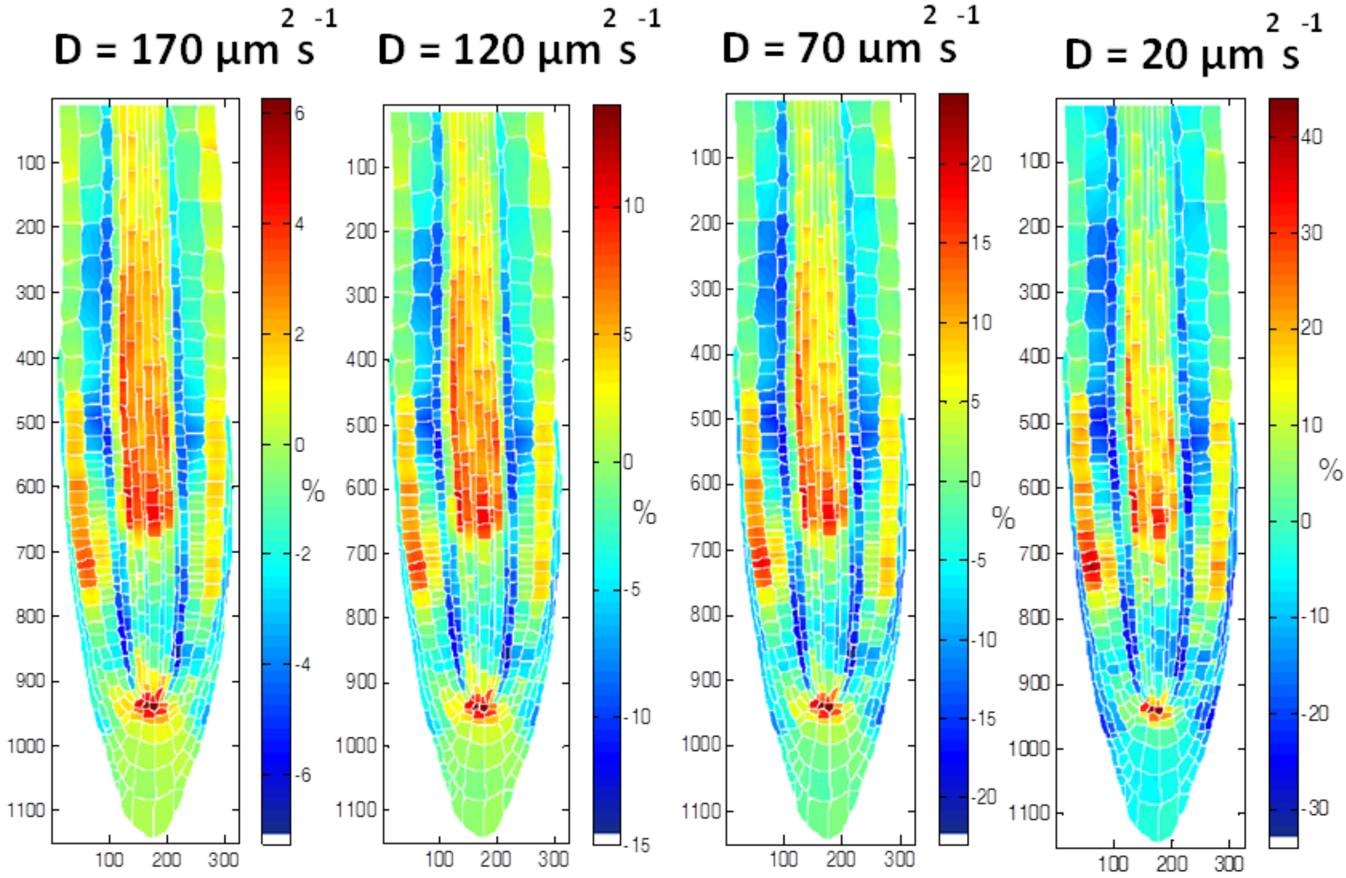

Figure S13. Effects of reducing auxin diffusion constant in cell wall on auxin patterning. This figure shows percentage difference from wildtype cytosolic auxin concentrations for auxin diffusion constants in the cell wall of 170, 120, 70 and 20 $\mu m^2 s^{-1}$. Wildtype diffusion rate = 220 $\mu m^2 s^{-1}$.

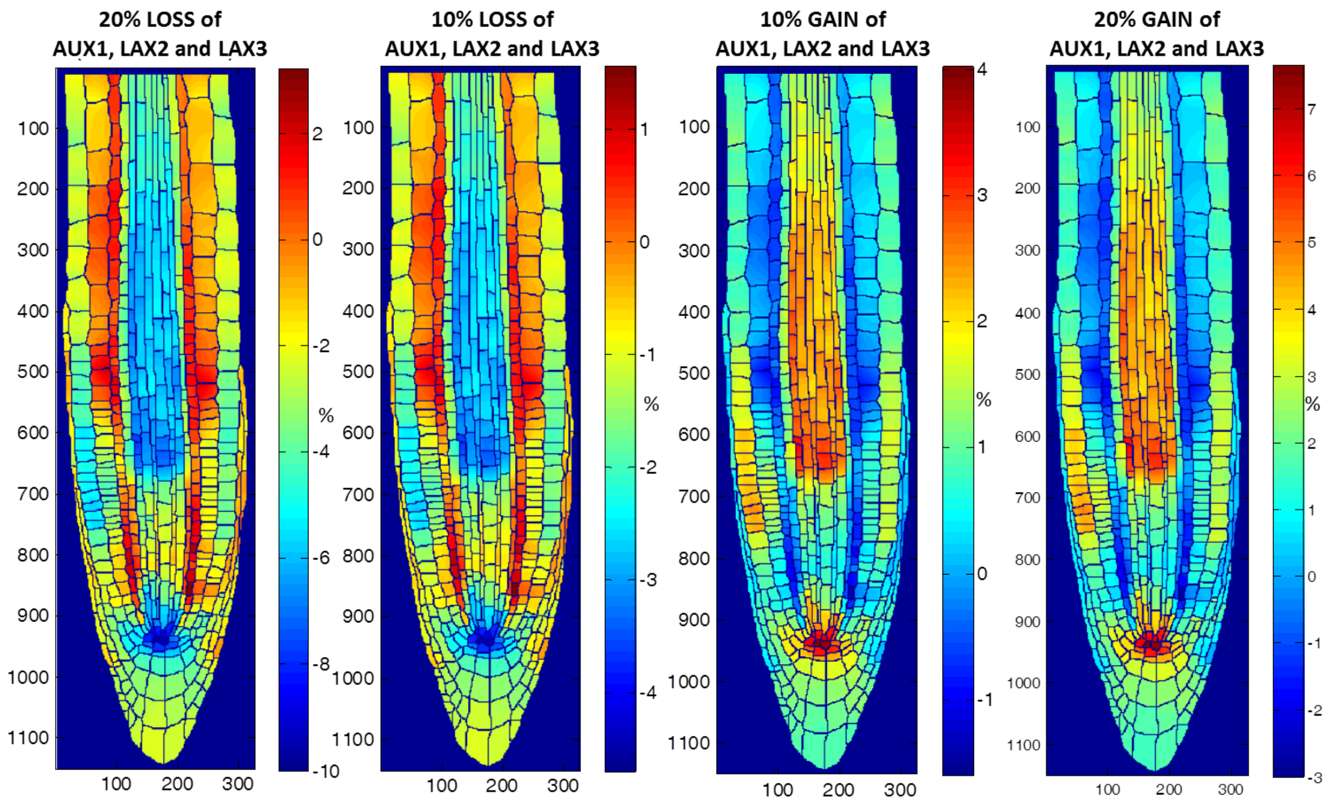

Figure S14. Effects of changing auxin influx levels on auxin patterning. This figure shows percentage difference from wildtype cytosolic auxin concentrations for changes in the concentrations of the AUX1, LAX2 and LAX3 auxin influx carriers. From left to right, the AUX1, LAX2 and LAX3 carrier concentrations are all adjusted by -20%, -10%, +10% or +20%, respectively.

A data-driven mechanistic model for studying the control of auxin patterning in Arabidopsis root development

The data-driven mechanistic model developed in this work integrates actual cell geometries, the level and polar or nonpolar localisation of auxin influx and efflux carriers, with a variety of experimental data about hormonal crosstalk, as described below.

1. Root structure with actual cell geometries, polar localisation of efflux carriers and nonpolar localisation of influx carriers

The digitised root structure was created using an image (Figure 1A) downloaded from www.simuplant.org (Band et al., 2014) which was generated from stacks of confocal images of roots stained with propidium iodide to define root geometry and cell organization. Software (SurfaceProject) was developed (Band et al., 2014) to process the confocal image data to produce a 2D root structure (Figure 1B).

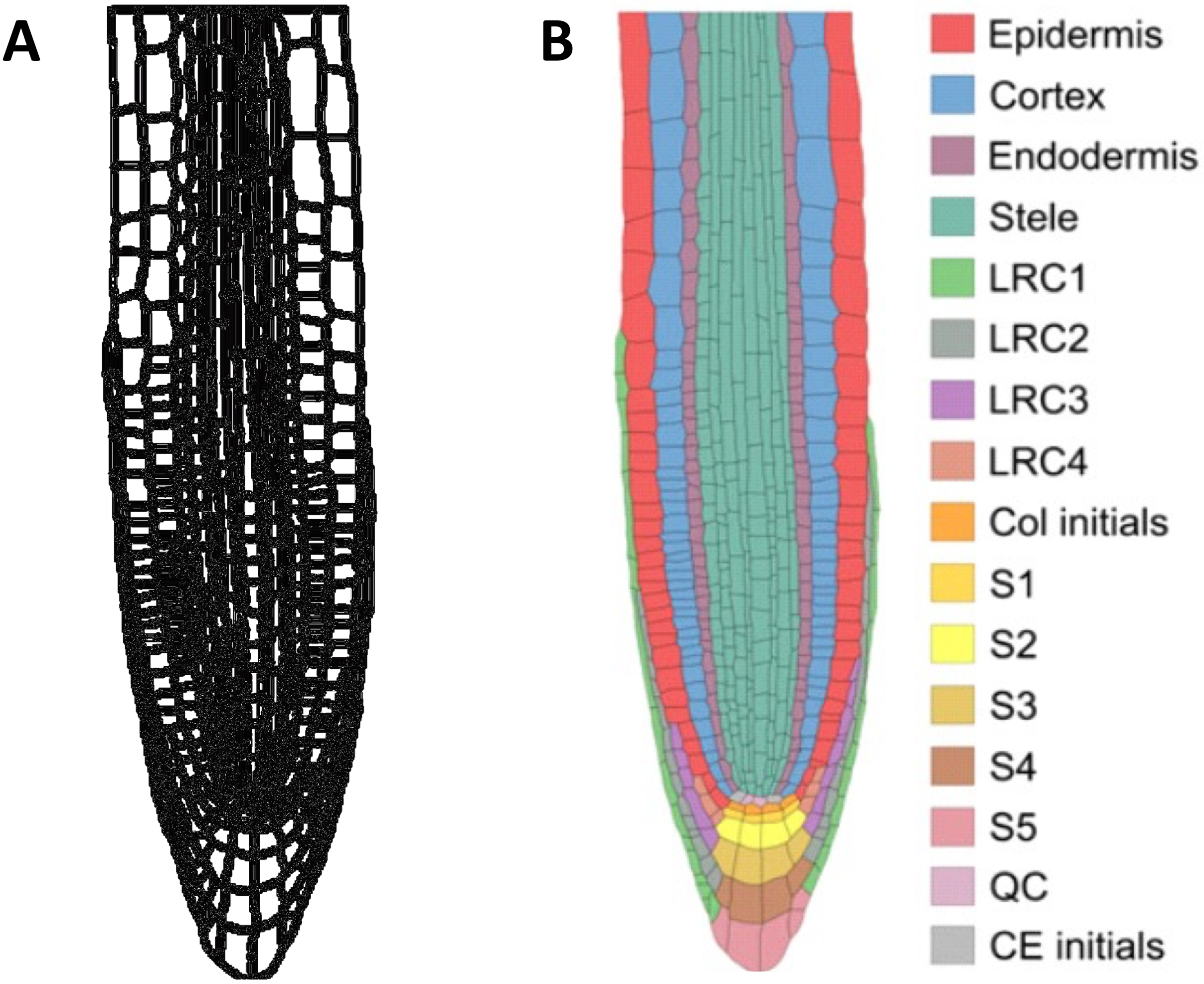

Figure 1: A. SimuPlant Image (Band et al., 2014); B. Root structure (Band et al., 2014). LRC - lateral root cap; S1 to S5 - columella; CE cortical endodermis; QC quiescent centre.

The initial downloaded SimuPlant image (Figure 1A) was scanned with ImageJ and the grid point data saved as an Excel file. The scanned image contained multiple imperfections and discontinuities in the

cell wall structures. To correct these, the file was processed by a series of error-checking MATLAB programs. The digital image was first searched for discontinuities in the cell walls which were corrected by interpolation from the end of a wall along points of weak ImageJ signal until another wall point was encountered. The resulting image was searched again for abnormal groups of cell wall grid points (GPs) and discontinuities, which were identified and removed. These processes were repeated several times since it was possible for the correction of one type of error to create another. The resulting file was formatted to highlight the cell walls to allow a visual check of the digitised root for remaining abnormalities or discontinuities. Finally all cytosolic GPs were set to 0 and cell wall GPs to 1, with GPs outside the root set to 9 and then the image was visually checked against the 2D map from Band et al., 2014 (Figure 1B). The next step was to create an individual cell wall for each cell. This was achieved by duplicating all cell wall GPs and then replacing the double wall at the exterior of the root with a single wall. The resulting root image was again searched for larger blocks of cell wall GPs which were each individually checked and corrected if necessary. The final image was visually checked for any wall discontinuities or abnormalities and a few manual adjustments made. In the basic root map, some cell wall GPs were adjacent to cytosolic points, having a nearest neighbour (NN) cytosolic point to the N, S, E or W. Other wall GPs did not have a NN cytosolic point, especially at multiple cell junctions, and could therefore be regarded as forming an extra-cellular space. The above process resulted in a basic digital root map which required further properties to be assigned to each GP for efficient use in the model. For example it would be necessary to define polarised efflux and non-polar influx carrier properties to the plasma membrane in selected cells. To allow automation of these steps, all cytosolic points were assigned cell numbers to allow automated identification of specific cells (Table 1) and apical, basal, and inner and outer lateral cell faces were defined for each cell. The final root map matrix contains 374,900 grid points defining 501 cells.

Table 1: Cell Numbers

	**Description of cell type (Band et al., 2014 Fig 1A)**	**Total number of individual cells (left, right sides)**	**Available number range for cells at right side of root**	**Available number range for cells at left side of root**
1	QC	2	100-104	NA
2	Cortical endodermal (CE) initials	2	105-109	NA
3	Columella initials	4	100-119	NA
4	Columella S1	4	120-129	NA
5	Columella S2	4	130-139	NA
6	Columella S3	4	140-149	NA
7	Columella S4	4	150-159	NA
8	Columella S5	5	160-169	NA
9	Lateral Root Cap (LRC1)	12, 16	200-219	220-229
10	LRC2	12,14	250-269	270-289
11	LRC3	8,5	300-319	320-339
12	LRC4	3,3	350-369	370-389
13	Epidermis	28,31	500-549	550-599
14	Cortex	38,41	600-649	650-699
15	Endodermis	39,42	700-749	750-799
16	Pericycle	23,33	800-849	850-899
17	6 Vascular cell files			
	(a) Outer pair	21,26	900-949	950-999
	(b) Middle pair	23,22	1000-1049	1050-1099
	(c) Inner pair	19,16	1100-1149	1150-1199
	**TOTAL NUMBER OF CELLS**	**501**		

Once the digital root map had been defined, with an individual cell wall structure for each cell and unique cell numbers (Figure 2A), it was necessary to assign the levels and localisation of the polar PIN efflux carriers and the non-polar AUX1/LAX influx carriers to the plasma membrane (included in the cell wall properties). PIN1 and 2 carrier levels are regulated in exactly the same way by the crosstalk network which also regulates the rate that the cytosolic PIN proteins are placed and removed from the plasma membrane, as previously described (Moore et al., 2015). Since the model does not differentiate between PIN1 and PIN2 (apart from polarity localisation), for the purposes of this section PIN1 and 2 will be jointly referred to as PIN12. Depending on the location in the root, the cell wall GPs are assigned different properties to define the rate of transfer of the PIN12 proteins from the cytosolic GPs to the neighbouring plasma membrane (Moore et al., 2015). The PIN12 transfer rates (low, medium or high) were based on experimental images from various publications. It was assumed that PIN12 are differentially expressed everywhere in the root tip (Moore et al., 2015), therefore as a default, all PIN12 transfer rates to the plasma membrane were initialised at a low level and then transfer rates at selected cell faces were reset to medium or high based on experimental images. Figure 2B summarises the transfer rate settings for PIN12 which define PIN12 polarity (1 = Blue/Low, 2 = Green/Medium, 3 = Red/High).

PIN12 transfer rates in the lateral root cap cells were set at medium (Green) on the apical cell faces from approximately the 200 level from the distal end of the root (Figure 2B) up to approximately the 325 level after which they were set to high (Red) on the apical cell faces (Laskowski et al., 2008). In the epidermal cells, the transfer rates were left at low (Blue) on all cell faces for the first 3 cells apical to the QC. For the next 3 cells they were set at medium on the apical faces and low on the lateral and basal faces, and thereafter set at high on the apical face and low on the lateral and basal faces (Laskowski et al., 2008; Muller et al., 1998). For the cortical cell files for the first 3 cells apical to the QC the transfer rates were set to low at all faces. For the next 3 cells the rates were set to medium on the basal face and low on the inner and outer lateral and apical faces. The rates were then set to high on the basal face, medium on the outer lateral and low on the inner lateral and apical faces through the meristematic zone (MZ) to approximately the 650 level. As the cells move out of the MZ there is a basal to apical shift and the transfer rates were then set at high on the apical cell face, medium on the outer lateral face and low on the basal and inner lateral faces (Kleine-Vehn et al., 2008; Muller et al., 1998). The outer lateral settings in the cortical cells were based on Figure 1D from Kleine-Vehn et al., 2008. In the vascular cylinder and pericycle, the transfer rates were set to high at the basal face of all cells and high on the inner lateral of the pericycle cells (Friml et al., 2002) while the rates at the remaining vascular and pericycle cell faces were set at low. The transfer rates were set at medium on the basal face and at low on the inner and outer lateral and apical faces of the endodermis cells (Friml et al., 2002).

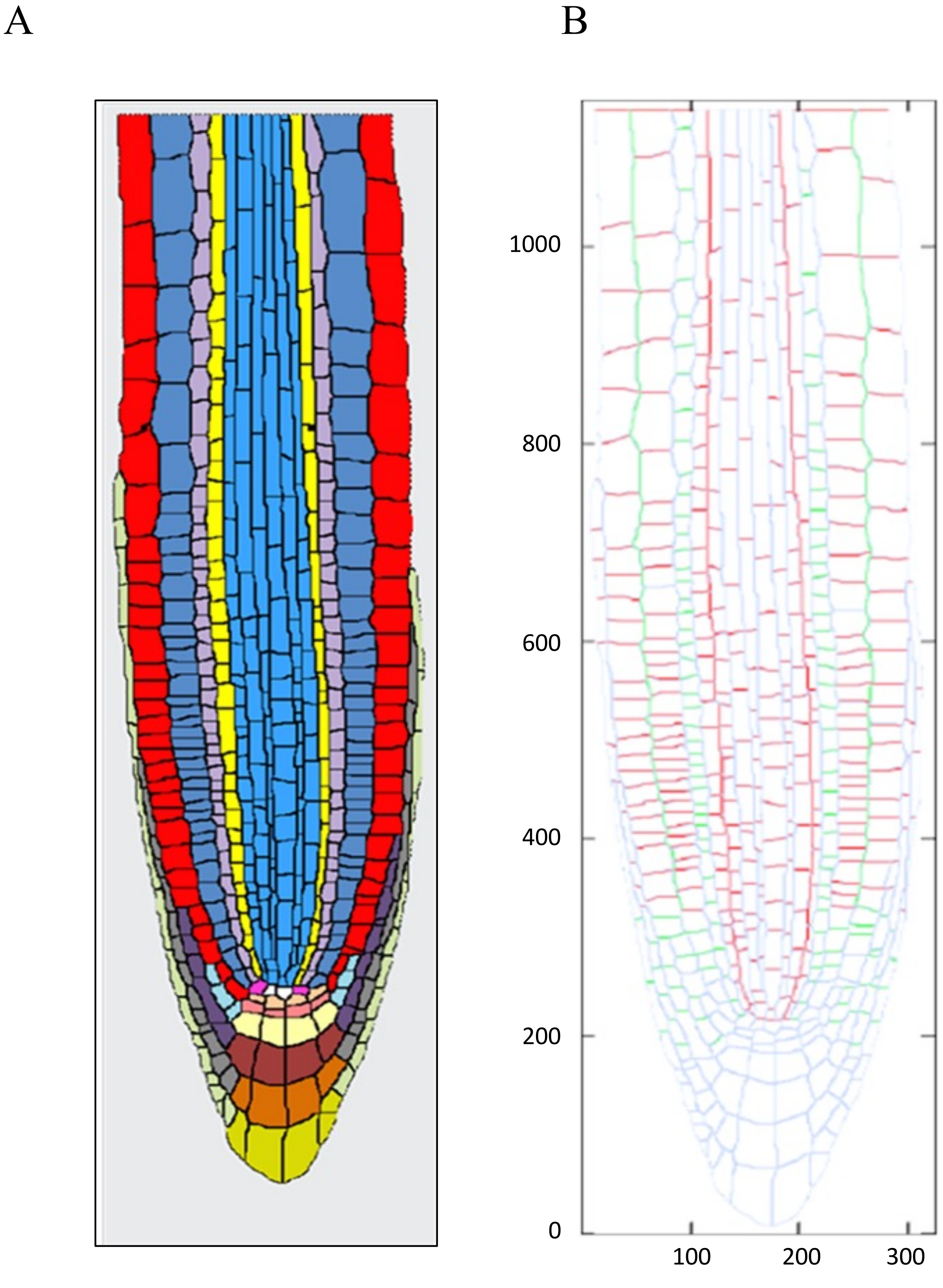

Figure 2A: Digital realistic root map. B: PIN12 transfer rates to the plasma membrane (RED = High, GREEN = Medium, BLUE = Low).

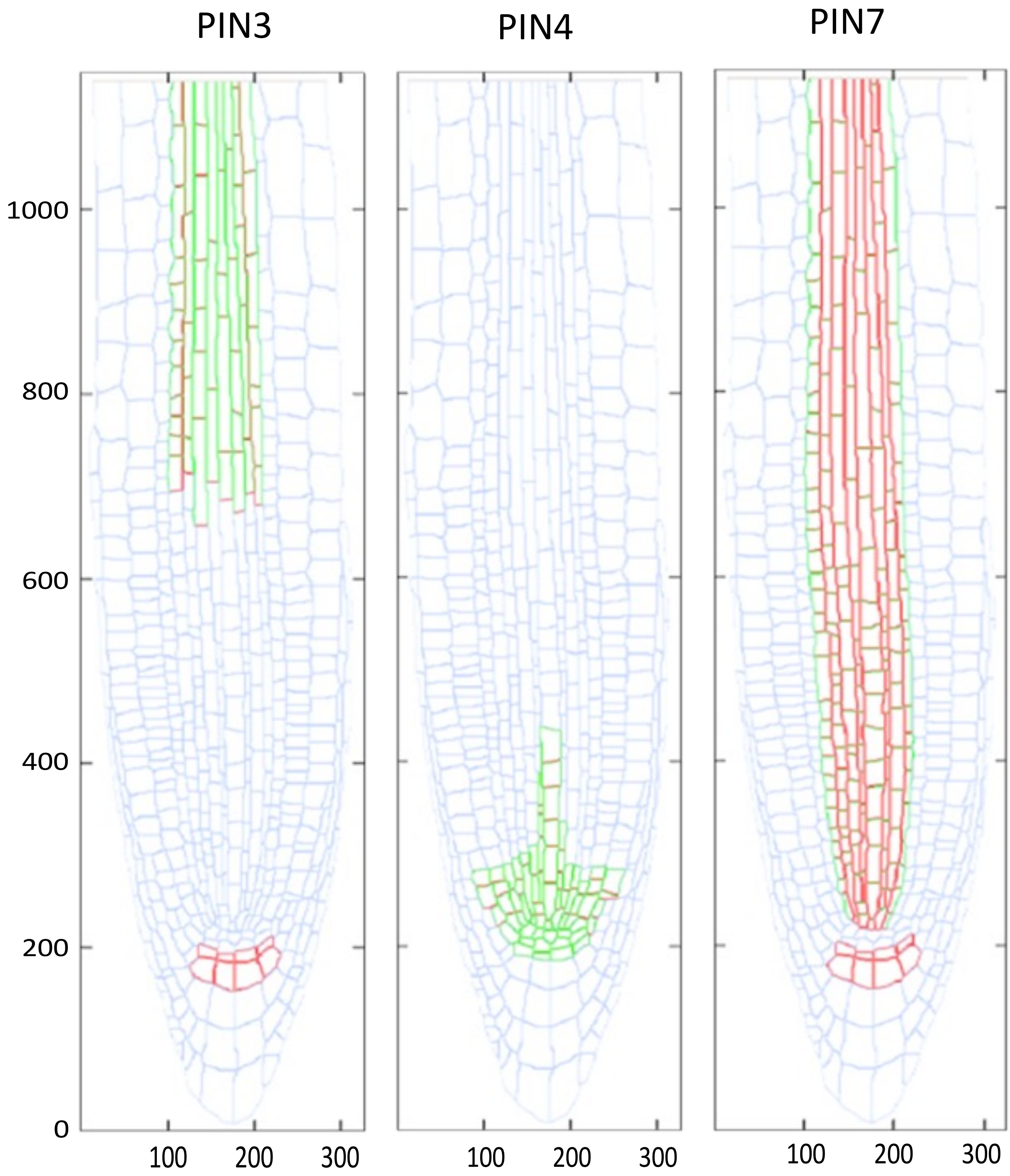

Figure 3 Efflux carrier WT concentration settings. PIN3: Red = 1.0μM, Green = 0.25μM, Blue = 0.06 μM. PIN4: Red = 0.5μM, Green = 0.1 μM, Blue = 0.02 μM. PIN7: Red = 1.0 μM, Green = 0.25 μM, Blue = 0.06 μM.

The PIN3, PIN4 and PIN7 efflux carrier concentration levels and polar localisation are not regulated by the network but have prescribed concentrations at selected cell faces based on experimental imaging from the literature (Blilou et al., 2005) with concentrations levels adjusted to produce WT auxin patterning (Figure 3). The model is set up with 4 possible concentration levels (however only 3 are used) for each efflux carrier for which concentrations are assigned by the user at model run time so that concentrations can be easily adjusted when searching for WT auxin patterning. PIN3 has non-polar localisation at a high level in the columella S1 and S2. It is localised at a high level at the basal face and at a medium level at the inner and outer lateral and apical faces of the vascular cells in the elongation zone (EZ). In the pericycle cells in the EZ, it has a high level of localisation at the inner lateral and basal faces and a medium level at the outer lateral and apical faces (Figure 3). It has non-polar localisation at a low level in all other cells. PIN4 has non-polar localisation at a medium level in the QC and initials and their immediate neighbours. In the other cells in the distal meristematic zone (MZ) it is localised at a high level at the basal face and at a medium level at the apical and inner and outer lateral faces. In all other cells, PIN4 has non-polar localisation at a low level at all cell faces (Figure 3). PIN7 is localised at a medium or high level at all cell faces in the pericycle and vascular cells in the MZ and EZ and in certain columella cells. In the pericycle cells it is localised at a high level on the inner lateral and basal faces and at a medium level at the apical and outer lateral faces. In the vascular cells it is localised at a high level at the inner and outer lateral and basal faces and at a medium level on the apical face. It is localised in a non-polar distribution at a high level at all faces of the columella S1 and S2 cells. At all other cells PIN7 has non-polar low level localisation (Figure 3).

The non-polar localisation of the auxin influx carriers AUX1, LAX2 and LAX3 (Figure 4) is again based on experimental imaging (Band et al., 2014) with concentrations adjusted to achieve WT auxin patterning. The model allows 15 possible concentration levels for AUX1, 8 possible levels for LAX2 and 4 levels for LAX3 (however only 3 were used for each carrier to define WT), and specific concentrations are assigned to each level by the user at model run time. AUX1 is localised at a medium level in the lateral root cap, at a medium level in the EZ and apical MZ of the epidermis, and at a medium level in the cortical cells in the EZ. It is localised at a medium level in the cortical and epidermal cells just apical to the QC and at a high level in the columella S1-S4. In all other cells AUX1 is localised at a low level. LAX2 is localised at a high level in the vascular and pericycle cells in the mid to distal region of the MZ, and in the QC and columella initials. There is zero LAX2 concentration in all other cells. LAX3 is localised at a high level in the columella S2 and has a zero concentration in all other cells.

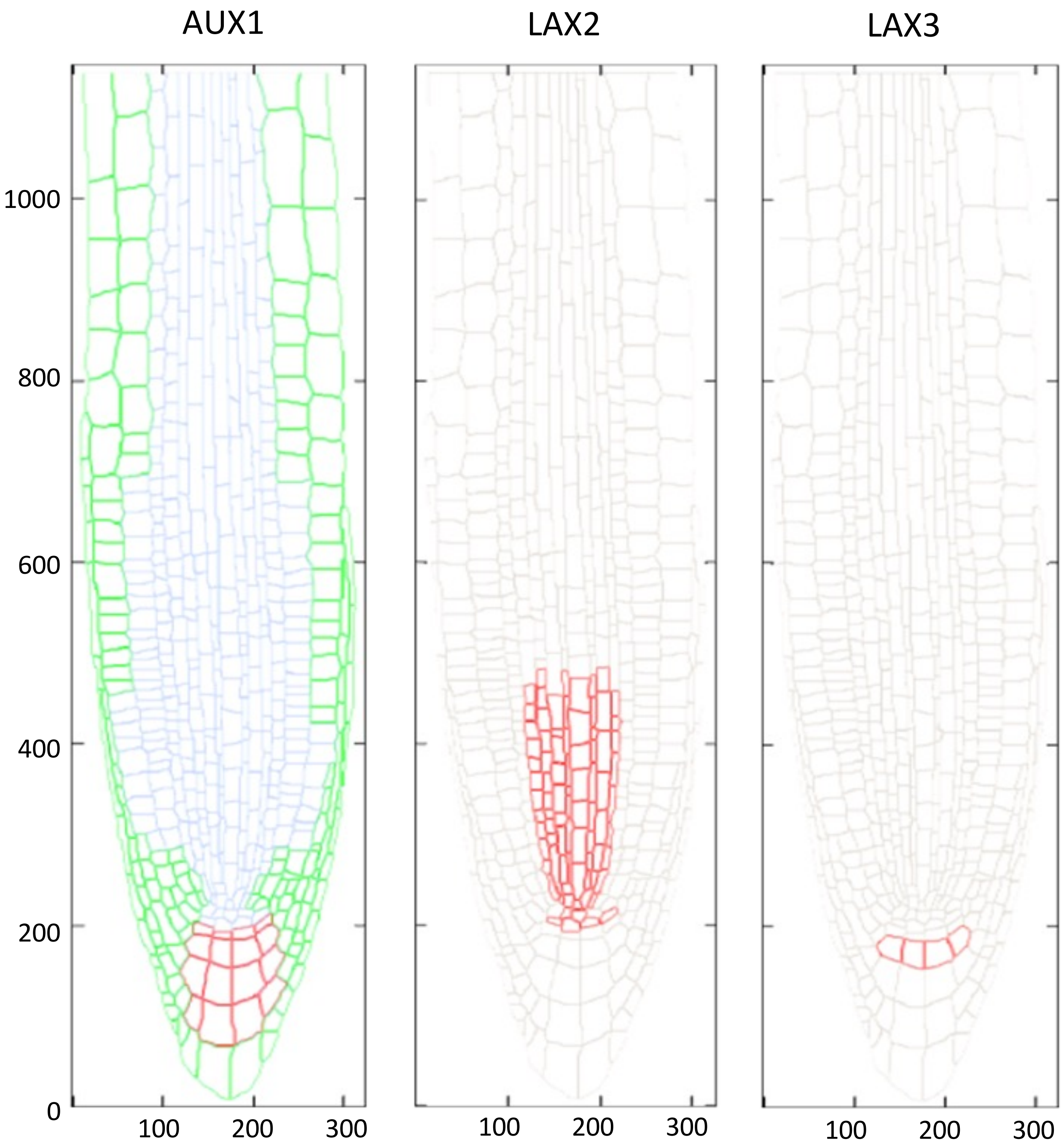

Figure 4 Influx carrier WT concentration settings. AUX1: Red/High = 2.25µM, Green/Medium = 1.0 µM, Blue/Low = 0.75 µM. LAX2: Red/High = 1.75 µM, Green/Medium = 0.0 µM, Blue/Low = 0.0 µM. LAX3: Red/High = 1.75 µM, Green/Medium = 0.0 µM, Blue/Low = 0.0 µM. Grey indicates zero concentration.

2. Hormonal crosstalk between PIN1,2 and three hormones (auxin, ethylene and cytokinin)

Crosstalk between auxin, ethylene, cytokinin and PIN1 and PIN2 was previously described using a network (Liu et al., 2010, 2013; Moore et al., 2015), which was constructed by iteratively combining modelling and experimental measurements. In previous research (Moore et al., 2015), we considered that AUX1 activity is positively regulated by the downstream ethylene signalling based on experimental observation (Figure 7B in Ruzicka *et al.*, 2007). Model results for AUX1 patterning (Fig. S12 in Moore et al., 2015) are in part similar to experimental imaging (Fig. S8 in Band *et al.* 2014) with AUX1 levels increasing proximally in the epidermis, and higher AUX1 levels in the outer cell layers compared to the central cell cylinder. Experimentally, it has been shown that, within the epidermis, AUX1 is present mainly in the elongation zone cells (Band *et al.* 2014). However, the model does not exhibit the elevated experimental AUX1 levels in the columella and near the QC or the proximally declining AUX1 levels in the central cylinder. We concluded that the differences between modelling and experimental results may indicate that, in addition to ethylene, other effectors may also regulate AUX1 activity (Moore et al., 2015). Therefore, we consider that the crosstalk between AUX1 and three hormones (auxin, ethylene and cytokinin) cannot be fully established so that AUX1 patterning can be fully predicted using the model. Thus, in this research, AUX1 localisation is prescribed using experimental data.

Although the regulatory relationships between auxin, ethylene, cytokinin and polar PIN1 and PIN2 proteins were previously established by iteratively combining experimental measurements with modelling analysis (Liu et al., 2010, 2013; Moore et al., 2015; Rowe et al., 2016), it is currently not possible to construct the crosstalk network between the three hormones and other auxin carriers, due to insufficient data and crosstalk complexity. For example, experimental and modelling analysis has suggested that spatial expression patterning of the influx carrier LAX3 is affected by expression of the efflux carrier PIN3 (Peret et al., 2013). However, other modelling and experimental analysis suggests that the induction of *PIN3* is not required to explain the switch-like expression of *LAX3* (Mellor et al., 2015). The feedback of GH3, which is an important component in the auxin-degradation pathway, may also have a role in *LAX3* expression (Mellor et al., 2016). Furthermore, a recent study has shown that the regulation of *PIN3* and *PIN7* expression by auxin and cytokinin in root development follows different mechanisms (Lavenus et al., 2016; Wang et al., 2015). These examples show the complexity of crosstalk between auxin, its carriers and other hormones.

In addition, extensive examination of published experimental data reveals that it is currently not possible to construct a network between the three hormones and other carriers, due to insufficient data and the complexity of crosstalk between hormones and auxin carriers. Therefore PIN3,4,7, AUX1 and LAX2 and 3 localisation is prescribed using experimental data.

Therefore, in the model developed in this work, the following crosstalk network (Figure 5) controls metabolism of two efflux carriers (PIN1and PIN2) and the hormones auxin, ethylene and cytokinin in the cytosolic spaces. The network allows quantitative description of PIN1 and PIN2 regulation by the three hormones and enables study of the relationship between auxin, PIN1 and PIN2 patterning.

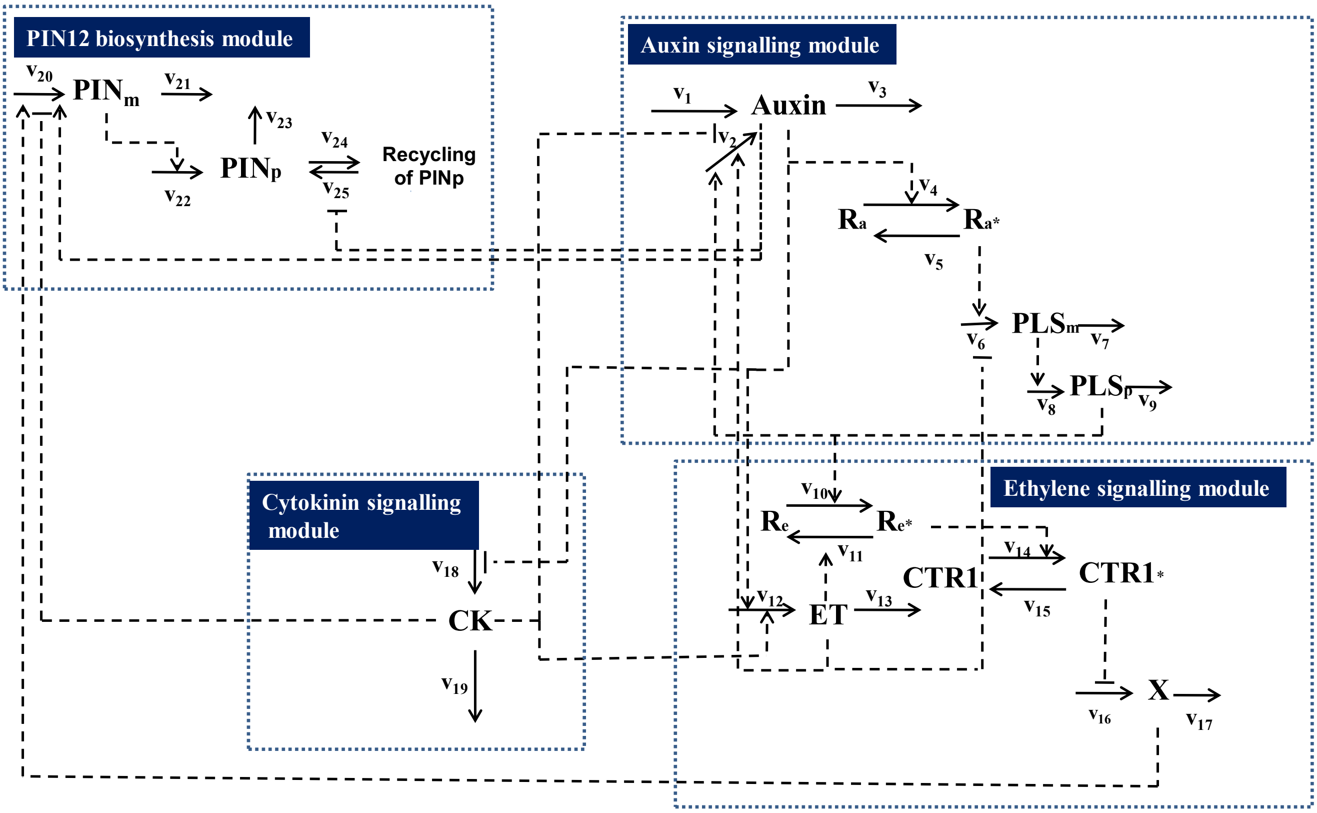

Figure 5: Hormonal crosstalk and gene expression network between PIN1,2 and three hormones (auxin, ethylene and cytokinin). Symbols: Auxin: Auxin hormone, ET: ethylene, CK: Cytokinin, PINm: PIN mRNA, PINp: PIN protein, PLSm: POLARIS mRNA, PLSp: POLARIS protein, X: Downstream ethylene signalling, Ra*: Active form of auxin receptor, Ra: Inactive form of auxin receptor, Re*: Active form of ethylene receptor, ETR1. Re: Inactive form of ethylene receptor, ETR1, CTR1*: Active form of CTR1, CTR1: Inactive form of CTR1.

3. A model that integrates actual cell geometries, the level and polar or nonpolar localisation of auxin influx and efflux carriers, with a variety of experimental data about hormonal crosstalk

The current research develops a model integrates actual cell geometries, the level and polar or nonpolar localisation of auxin influx and efflux carriers, with a variety of experimental data about hormonal crosstalk. We note that our previous research (Moore et al., 2015) did not include LAX2,3 and PIN3,4,7, and it used a rectangular root structure that does not describe realistic cellular geometries. Moreover, the rectangular root structure used in our previous research (Moore et al., 2015) does not have a root cap, and it does not include extracellular space. The model developed in the current research integrates a root structure with actual cell geometries, the level and polar or nonpolar localisation of all auxin influx (AUX1, LAX2,3) and efflux carriers (PIN1,2,3,4,7), with a variety of experimental data about hormonal crosstalk. Since the ABCB family of auxin carriers can reversibly redirect auxin flux, the role of ABCB transporters has been incorporated into PIN and AUX1/LAX activity to simplify modelling analysis. Therefore, the current research has integrated all known important auxin transporters for cell to cell communication with a wide range of experimental data about the crosstalk between PIN1,2 and three hormones (auxin, ethylene and cytokinin) (Liu et al., 2010, 2013; Moore et al., 2015 and references therein).

Kinetic equations and parameters for crosstalk between PIN1, PIN2, auxin, ethylene and cytokinin and for the recycling of PIN1 and PIN2 are as previously described (Moore et al., 2015). Concentration levels for PIN3,4,7 and AUX1, LAX2,3 are adjusted so that the model reproduces key features of wild-type auxin patterning, as shown in Figures 1-4 in this Supplemental Methods.

References

Band, L. R. *et al*. Systems analysis of auxin transport in the Arabidopsis root apex. *Plant Cell* **26**, 862–875 (2014).

Blilou, I. *et al*. The PIN auxin efflux facilitator network controls growth and patterning in Arabidopsis roots. *Nature* **433***,* 39-44 (2005).

Friml, J. *et al*. AtPIN4 mediates sink-driven auxin gradients and root patterning in Arabidopsis. *Cell* **108**, 661–673 (2002).

Kleine-Vehn, J. *et al*. Cellular and molecular requirements for polar PIN targeting and transcytosis in plants. *Mol. Plant* **1***,*1056-1066 (2008).

Laskowski, M. *et al*. Root system architecture from coupling cell shape to auxin transport. *PLoS Biol.*, **6** (2008).

Lavenus, J., Guyomarc'h, S. & Laplaze, L. PIN transcriptional regulation shapes root system architecture. *Trends Plant Sci*. **21**, 175-177 (2016).

Liu, J. L., Mehdi, S., Topping, J., Tarkowski, P. & Lindsey, K. Modelling and experimental analysis of hormonal crosstalk in Arabidopsis. *Molec. Syst. Biol.* **6**, 373 (2010).

Liu, J. L., Mehdi, S., Topping, J., Friml, J. & Lindsey, K. Interaction of PLS and PIN and hormonal crosstalk in Arabidopsis root development. *Front. Plant Sci.* **4**, 75 (2013).

Mellor, N. *et al*. Modelling of Arabidopsis LAX3 expression suggests auxin homeostasis. J. Theor. Biol. **366**, 57–70 (2015).

Mellor, N., Bennett, M.J. & King, J.R. GH3-mediated auxin conjugation can result in either transient or oscillatory transcriptional auxin responses. Bull. Math. Biol. **78**, 210-34 (2016).

Moore, S. *et al*. Spatiotemporal modelling of hormonal crosstalk explains the level and patterning of hormones and gene expression in Arabidopsis thaliana wildtype and mutant roots. *New Phytol.* **207**, 1110–1122 (2015).

Müller, A. *et al*. AtPIN2 defines a locus of Arabidopsis for root gravitropism control. *EMBO J.* **17**, 6903-6911 (1998).

Rowe, J. H., Topping, J. F., Liu, J. & Lindsey, K. Abscisic acid regulates root growth under osmotic stress conditions via an interacting hormonal network with cytokinin, ethylene and auxin. *New Phytol.* **211**, 225-239 (2016).

Ruzicka, K. *et al*. Ethylene regulates root growth through effects on auxin biosynthesis and transport-dependent auxin distribution. *Plant Cell* **19**, 2197–2212 (2007).

Wang, H.-Z. *et al.* Transcriptional regulation of *PIN* genes by FOUR LIPS and MYB88 during Arabidopsis root gravitropism. *Nat. Commun*. **6**, 8822 (2015).
